# Structural and Biochemical Characterization of *Mycobacterium tuberculosis* Zinc SufU-SufS Complex

**DOI:** 10.3390/biom13050732

**Published:** 2023-04-24

**Authors:** Ingie Elchennawi, Philippe Carpentier, Christelle Caux, Marine Ponge, Sandrine Ollagnier de Choudens

**Affiliations:** 1 CNRS, CEA, IRIG, Laboratoire de Chimie et Biologie des Métaux, Université Grenoble Alpes, 38000 Grenoble, France; 2 European Synchrotron Radiation Facility, 38000 Grenoble, France

**Keywords:** iron-sulfur, SufS, SufU, zinc-containing protein, *Mycobacterium tuberculosis*, cysteine desulfurase, sulfurtransferase, protein-protein interaction, protein complex

## Abstract

Iron-sulfur (Fe-S) clusters are inorganic prosthetic groups in proteins composed exclusively of iron and inorganic sulfide. These cofactors are required in a wide range of critical cellular pathways. Iron-sulfur clusters do not form spontaneously in vivo; several proteins are required to mobilize sulfur and iron, assemble and traffic-nascent clusters. Bacteria have developed several Fe-S assembly systems, such as the ISC, NIF, and SUF systems. Interestingly, in *Mycobacterium tuberculosis* (*Mtb*), the causative agent of tuberculosis (TB), the SUF machinery is the primary Fe-S biogenesis system. This operon is essential for the viability of *Mtb* under normal growth conditions, and the genes it contains are known to be vulnerable, revealing the *Mtb* SUF system as an interesting target in the fight against tuberculosis. In the present study, two proteins of the *Mtb* SUF system were characterized for the first time: Rv1464(*sufS*) and Rv1465(*sufU*). The results presented reveal how these two proteins work together and thus provide insights into Fe-S biogenesis/metabolism by this pathogen. Combining biochemistry and structural approaches, we showed that Rv1464 is a type II cysteine-desulfurase enzyme and that Rv1465 is a zinc-dependent protein interacting with Rv1464. Endowed with a sulfurtransferase activity, Rv1465 significantly enhances the cysteine-desulfurase activity of Rv1464 by transferring the sulfur atom from persulfide on Rv1464 to its conserved Cys40 residue. The zinc ion is important for the sulfur transfer reaction between SufS and SufU, and His354 in SufS plays an essential role in this reaction. Finally, we showed that *Mtb* SufS-SufU is more resistant to oxidative stress than *E. coli* SufS-SufE and that the presence of zinc in SufU is likely responsible for this improved resistance. This study on Rv1464 and Rv1465 will help guide the design of future anti-tuberculosis agents.

## 1. Introduction

**Figure 1:**
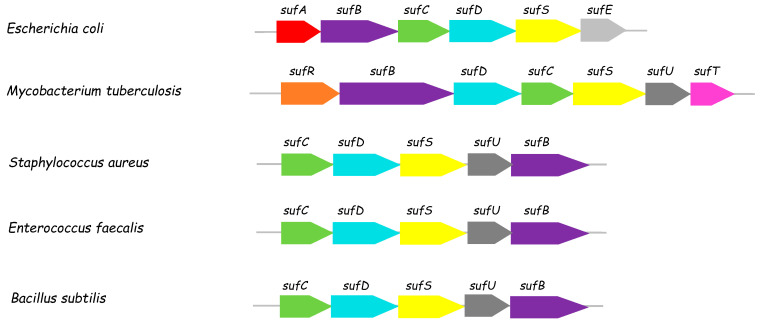
SUF operons in Gram-negative (*E. coli*), Gram-positive (*B. subtilis*), and some pathogenic bacteria (*S. aureus*, *Enterococcus faecalis*, and *M. tuberculosis*).

Iron-sulfur (Fe-S) clusters are inorganic prosthetic groups contained in proteins composed exclusively of iron and inorganic sulfide [1]. These cofactors are essential for a wide range of critical cellular pathways. Fe-S metalloproteins carry out diverse reactions, including electron transfer, substrate activation, and binding, but also act as environmental sensors and structural elements [2]. Due to the inherent toxicity of free iron and sulfide in the cellular environment, the conditions for the assembly of Fe-S clusters are not simple. Thus, several dedicated proteins are required to mobilize sulfur and iron during the assembly and trafficking of nascent Fe-S clusters. In eukaryotes, a large number of proteins are involved in Fe-S biogenesis. They are localized in several subcellular compartments, although the mitochondria play a central role in regulating Fe-S metabolism [3]. Three types of de novo Fe-S biogenesis systems have long been known in prokaryotes: the ISC, NIF, and SUF systems [4,5]. More recently, two minimal Fe-S cluster biogenesis machineries encoding ISC-like and SUF-like components were also described: MIS (Minimal Iron-Sulfur) and SMS (Suf Minimal System), respectively [5]. The ISC system is considered to be the housekeeping system, whereas the SUF system acts as a backup system under stress conditions, and the NIF system specializes in the maturation of nitrogenase in N_2_-fixing bacteria. Although their components differ, the NIF, ISC, and SUF systems facilitate Fe-S cluster biogenesis according to the same basic principles: a cysteine desulfurase takes sulfur from L-cysteine and transfers it as a persulfide onto a scaffold protein, which also receives Fe^2+^ and electrons to build an Fe-S cluster. Carrier proteins then transfer the cluster to apo-protein targets [4]. Interestingly, *Mycobacterium tuberculosis (Mtb),* the causative agent of tuberculosis (TB) that still kills 1.5 billion people annually, contains a single gene cluster with homology to the SUF system, Rv1460(*sufR*), Rv1461(*sufB*), Rv1462(*sufD*), Rv1463(*sufC*), Rv1464(*sufS*), Rv1465(*sufU*), and Rv1466(*sufT*) (Figure 1) [6,7]. Among these genes, Rv1464 is predicted to be a cysteine-desulfurase enzyme, providing sulfur from L-cysteine for Fe-S formation. The *Mtb* genome encodes another gene (Rv3025c) sharing homology with cysteine-desulfurase enzymes, that may also be involved in sulfur mobilization for Fe-S cluster assembly in this microorganism. The Rv3025c gene is present outside the *suf* locus and corresponds to a separate ORF. It encodes the cysteine-desulfurase IscS and is not surrounded by other isc genes [6], suggesting that it is not involved in Fe-S biogenesis. It may be involved in other (Fe-S-independent) pathways, such as the thiolation of tRNA molecules (thiouridine) and thiamin biosynthesis [8]. Thus, Rv1464 and genes belonging to the suf operon appear to constitute the primary Fe-S biogenesis system in *Mtb*. This operon was shown to be essential for *Mtb* viability under normal growth conditions [6,9,10].

Recently, some genes in the *suf* operon were shown to be vulnerable [11], identifying the *Mtb* SUF system as an interesting target in the fight against tuberculosis [12].

Over the last two decades, the *Escherichia coli* and *Bacillus subtilis* SUF systems have been intensively investigated. For the *E. coli* system (*sufABCDSE*), three main findings were established. First, the SufS-SufE proteins provide sulfur from L-cysteine through the SufS enzyme’s cysteine-desulfurase activity. The activity of this enzyme depends on the participation of the sulfurtransferase protein SufE, mediating the transfer of the protected persulfide sulfur from SufS to SufB [13,14,15,16] when in complex with SufD and/or SufC. Second, the SufB, SufC, and SufD proteins form a SufBC_2_D complex that acts as a scaffold for the assembly of Fe-S clusters [17,18,19]. Third, the SufA protein is an Fe-S transporter, transferring the cluster from the SufBC_2_D complex to cellular targets [17,20].

In *B. subtilis* Gram(+) bacteria, the *suf* operon (*sufCDSUB*) is slightly different from that in Gram(−) bacteria such as *E. coli* (Figure 1). Instead of SufE, it codes for a SufU protein, which bears more similarity to NifU and IscU scaffold proteins than SufE, although they share the same fold. After recombinant expression in *E. coli*, *B. subtilis* SufU contains one zinc ion per monomer and interacts with SufS [21,22]. *B. subtilis* SufU can act as a sulfur acceptor substrate of SufS and is also reported to play a role as an Fe-S scaffold since it can enhance the reconstitution of the eukaryotic Leu1 protein [22,23]. In contrast, FeS-SufU cannot promote activation of the SufS cysteine-desulfurase activity [23]. Recently, the crystal structure of the *B. subtilis* Zn-SufU-SufS complex was solved at 2.3 Å resolution, revealing the importance of SufU-SufS binding for the transfer of sulfur from SufS to SufU [24]. The *B. subtilis* SufB, SufC, and SufD proteins have not yet been characterized due to the instability of SufB [25].

From its genetic organization, the *Mtb suf* operon (*sufRBDCSUT*) is more similar to the *B. subtilis* suf operon than the *E. coli* suf operon, as it contains a putative SufU protein (Figure 1). The *Mtb* SUF system has not yet been extensively studied, but it is attracting increasing interest from researchers seeking to develop new approaches to fight tuberculosis. Although SufB, SufC, and SufD have been shown to interact in cellulo [6], SufR is known to be an Fe-S regulator of the *suf* operon [26], and SufT was identified as an accessory factor in Fe-S biogenesis [27], the *Mtb* SufS, SufU, SufB, SufC, and SufD proteins remain to be functionally and structurally characterized. The purpose of the present study was to characterize two proteins from the *Mtb suf* operon, SufS and SufU, by biochemical and structural methods in order to discover how they interact and operate together. More generally, the objective was to gain new insights into *Mycobacterium tuberculosis* Fe-S biogenesis/metabolism, to help the future design of anti-tuberculosis agents.

## 2. Materials and Methods

### 2.1. Materials

All products were from Sigma-Aldrich unless otherwise indicated.

### 2.2. Plasmid Construction

A synthetic gene coding for *Mycobacterium tuberculosis* SufS was purchased from GenScript™. The DNA sequence was inserted into a pYUB28b plasmid by the EZ PCR cloning method (GenScript™) to produce a protein containing an N-ter 6xHis tag (violet; see below in the DNA and protein sequences) followed by a Tobacco Etch Virus (TEV) cleavage site (green; see below in the DNA and protein sequences). This construct corresponds to the pYUB28b-sufS plasmid (hygromycin resistance). The corresponding DNA sequence is as follows:

**ATG**CACCACCACCACCACCACGAGAATCTTTATTTTCAGGGC**ATG**ACGGCCTCGGTGAACTCGCTCGATCTGGCGGCGATTCGCGCCGATTT
CCCCATCCTCAAGCGCATCATGCGGGGTGGAAACCCGTTGGCGTATTTGGACTCCGGCGCCACCTCACAACGCCCGCTGCAGGTCCTCGACG
CCGAGCGCGAGTTCCTGACCGCGTCCAACGGCGCGGTCCATCGTGGCGCGCACCAGCTGATGGAGGAGGCGACCGACGCCTACGAGCAGGGC
CGCGCGGACATCGCGTTATTCGTCGGCGCCGACACGGACGAGCTGGTGTTCACCAAAAATGCCACCGAGGCGCTCAACCTGGTGTCATATGT
GCTGGGGGACAGCCGTTTCGAGCGTGCCGTCGGCCCCGGCGACGTGATCGTCACCACCGAGCTGGAGCATCACGCCAACCTGATCCCGTGGC
AGGAGCTGGCCCGGCGCACCGGGGCCACATTGCGCTGGTACGGGGTGACTGACGACGGGCGCATCGACCTGGACTCGCTGTATCTGGACGAC
CGTGTCAAAGTCGTTGCGTTCACCCATCATTCCAATGTGACCGGGGTGCTGACACCGGTGAGCGAGCTGGTCTCCCGCGCCCACCAGTCGGG
TGCGCTGACCGTGCTGGACGCCTGCCAGTCGGTGCCGCACCAGCCGGTTGACCTGCACGAACTCGGCGTCGACTTCGCCGCGTTTTCCGGAC
ATAAAATGCTGGGCCCCAACGGAATCGGTGTGCTGTACGGCCGCCGTGAGCTGCTAGCGCAGATGCCCCCATTTCTCACCGGCGGTTCGATG
ATCGAAACGGTGACCATGGAAGGCGCCACCTACGCGCCGGCGCCGCAACGGTTCGAGGCCGGTACCCCGATGACCTCCCAGGTGGTCGGGTT
GGCCGCCGCGGCCCGCTATCTCGGCGCGATCGGCATGGCCGCGGTGGAGGCCCACGAGCGGGAGCTGGTAGCCGCGGCCATCGAAGGCCTGT
CCGGCATCGACGGTGTGCGGATCCTTGGCCCGACGTCGATGCGGGACCGAGGGTCGCCGGTGGCGTTCGTCGTCGAGGGCGTGCACGCGCAC
GACGTGGGTCAGGTACTCGACGACGGCGGCGTGGCGGTGCGGGTCGGGCACCACTGCGCGCTGCCGCTGCACCGCAGGTTCGGTCTGGCCGC
CACCGCGCGGGCGTCGTTCGCGGTGTACAACACCGCAGACGAGGTGGACCGCTTGGTGGCCGGCGTGCGGCGATCCCGGCATTTCTTTGGAA
GAGCG**TGA**

it produces the following protein sequence (46.4 kDa):

**M**HHHHHHENLYFQG**M**TASVNSLDLAAIRADFPILKRIMRGGNPLAYLDSGATSQRPLQVLDAEREFLTASNGAVHRGAHQLMEEATDAYEQG
RADIALFVGADTDELVFTKNATEALNLVSYVLGDSRFERAVGPGDVIVTTELEHHANLIPWQELARRTGATLRWYGVTDDGRIDLDSLYLDD
RVKVVAFTHHSNVTGVLTPVSELVSRAHQSGALTVLDACQSVPHQPVDLHELGVDFAAFSGHKMLGPNGIGVLYGRRELLAQMPPFLTGGSM
IETVTMEGATYAPAPQRFEAGTPMTSQVVGLAAAARYLGAIGMAAVEAHERELVAAAIEGLSGIDGVRILGPTSMRDRGSPVAFVVEGVHAH
DVGQVLDDGGVAVRVGHHCALPLHRRFGLAATARASFAVYNTADEVDRLVAGVRRSRHFFGRA

After cleavage of the 6xHis by TEV, the protein has the following sequence and a size of 44.6 kDa:

GMTASVNSLDLAAIRADFPILKRIMRGGNPLAYLDSGATSQRPLQVLDAEREFLTASNGAVHRGAHQLMEEATDAYEQGRADIALFVGADTDELVFTKNATEALNLVSYVLGDSRFERAVGPGDVIVTTELEHHANLIPWQELARRTGATLRWYGVTDDGRIDLDSLYLDDRVKVVAFTHHSNVTGVLTPVSELVSRAHQSGALTVLDACQSVPHQPVDLHELGVDFAAFSGHKMLGPNGIGVLYGRRELLAQMPPFLTGGSMIETVTMEGATYAPAPQRFEAGTPMTSQVVGLAAAARYLGAIGMAAVEAHERELVAAAIEGLSGIDGVRILGPTSMRDRGSPVAFVVEGVHAHDVGQVLDDGGVAVRVGHHCALPLHRRFGLAATARASFAVYNTADEVDRLVAGVRRSRHFFGRA

The synthetic gene coding *Mycobacterium tuberculosis* SufU was purchased from GenScript™. The DNA sequence was codon-optimized for expression in *E. coli* and designed to be inserted into a pUC57 plasmid (Novagen™). The construct was digested with NdeI and XhoI and cloned into the pET15b vector at the same restriction sites to produce the pET15b-sufU plasmid (ampicillin resistance), generating a protein containing an N-ter 6xHis tag (violet; see below in the DNA and protein sequences) followed by a thrombin cleavage site (green; see below in the sequences). The DNA sequence inserted into the pUC57 vector is as follows:

**CATATG**GTTACGCTGCGTCTGGAGCAAATCTATCAGGACGTAATCCTGGACCATTACAAACATCCGCAGCACCGTGGTCTGCGTGAACCGTT
CGGCGCACAGGTTTACCACGTTAACCCGATCTGCGGTGATGAAGTTACTCTGCGTGTTGCCCTGTCTGAAGACGGTACCCGTGTTACTGACG
TTTCTTACGACGGTCAGGGTTGCAGCATTTCTCAGGCTGCTACTAGCGTTCTGACCGAGCAGGTTATTGGCCAGCGTGTGCCGCGTGCTCTG
AACATCGTCGACGCCTTCACTGAAATGGTGAGCTCTCGTGGTACCGTGCCGGGCGATGAGGATGTTCTGGGTGATGGCGTGGCGTTCGCAGG
TGTTGCAAAGTACCCAGCGCGTGTTAAGTGCGCACTGCTGGGTTGGATGGCTTTCAAAGATGCCCTGGCACAAGCGAGCGAAGCCTTCGAAG
AAGTCACGGACGAACGTAACCAACGCACCGGTTGA**CTCGAG**

(CATATG: NdeI, CTCGAG: XhoI)

The DNA sequence inserted into the pET15b vector is as follows:

**ATG**GGCAGCAGCCATCATCATCATCATCACAGCAGCGGCCTGGTGCCGCGCGGCAGCCATATGGTTACGCTGCGTCTGGAGCAAATCTATCA
GGACGTAATCCTGGACCATTACAAACATCCGCAGCACCGTGGTCTGCGTGAACCGTTCGGCGCACAGGTTTACCACGTTAACCCGATCTGCG
GTGATGAAGTTACTCTGCGTGTTGCCCTGTCTGAAGACGGTACCCGTGTTACTGACGTTTCTTACGACGGTCAGGGTTGCAGCATTTCTCAG
GCTGCTACTAGCGTTCTGACCGAGCAGGTTATTGGCCAGCGTGTGCCGCGTGCTCTGAACATCGTCGACGCCTTCACTGAAATGGTGAGCTC
TCGTGGTACCGTGCCGGGCGATGAGGATGTTCTGGGTGATGGCGTGGCGTTCGCAGGTGTTGCAAAGTACCCAGCGCGTGTTAAGTGCGCAC
TGCTGGGTTGGATGGCTTTCAAAGATGCCCTGGCACAAGCGAGCGAAGCCTTCGAAGAAGTCACGGACGAACGTAACCAACGCACCGGT**TGA**

which produces the following 20-kDa protein sequence:

MGSSHHHHHHSSGLVPRGSHMVTLRLEQIYQDVILDHYKHPQHRGLREPFGAQVYHVNPICGDEVTLRVALSEDGTRVTDVSYDGQGCSISQAATSVLTEQVIGQRVPRALNIVDAFTEMVSSRGTVPGDEDVLGDGVAFAGVAKYPARVKCALLGWMAFKDALAQASEAFEEVTDERNQRTG

After cleavage of the 6xHis tag with thrombin, the resulting 17.7-kDa protein has the following sequence:

GSHMVTLRLEQIYQDVILDHYKHPQHRGLREPFGAQVYHVNPICGDEVTLRVALSEDGTRVTDVSYDGQGCSISQAATSVLTEQVIGQRVPRALNIVDAFTEMVSSRGTVPGDEDVLGDGVAFAGVAKYPARVKCALLGWMAFKDALAQASEAFEEVTDERNQRTG

Plasmids were sequenced by Eurofins Genomics (Germany), and gene sequences were checked using CLC Sequence Viewer to ensure that no error had been introduced during the PCR reaction.

### 2.3. Protein Expression and Purification

SufS was expressed in a special T7-compatible *M. smegmatis* strain, mc^2^4517 [28,29], and similar to all mycobacteria, *M. smegmatis* should be cultured in a dedicated medium [30]. Electrocompetent *M. smegmatis* mc^2^4517 cells were prepared as described [29]. Briefly, *M. smegmatis* mc^2^4517 cells were grown at 37 °C with shaking in a 7H9 Middlebrook broth supplemented with 10% (*v*/*v*) ADC (Albumin, Dextrose, Catalase), 0.05% (*v*/*v*) Tween-80, 0.5% (*v*/*v*) glycerol, and 50 μg/mL kanamycin. Once the optical density (OD_600_) of *M. smegmatis* reached 0.5, cell cultures were transferred to an ice bath and incubated on ice for 1.5 h. Cells were then centrifuged and washed three times in 10% (*v*/*v*) ice-cold glycerol before incubating on ice once again for 1.5 h. Cells were finally resuspended in 10% (*v*/*v*) glycerol. The resulting competent *M. smegmatis* mc^2^4517 cells were transformed by electroporation with the pYUB28b-sufS vector. Each transformation reaction used 20 μL of competent cells and 1 μL pYUB28b-sufS plasmid DNA (400 ng). After a short incubation on ice, 280 μL ice-cold 10% (*v*/*v*) glycerol was added to the mixture in a pre-chilled standard gap electroporation cuvette. After a further 30 min incubation on ice, cells were electroporated using a Bio-Rad Gene Pulser^®^ II RF Module electroporator (R = 1000 Ω, Q = 25 µF, V = 2.5 kV). Immediately after electroporation, 1 mL of ice-cold 7H9/ADC/Tween80 was added to the cuvettes. Cuvettes were incubated on ice for 10–15 min before transferring cells to sterile Eppendorf tubes and allowing them to recover for 3 h at 37 °C with shaking. After recovery, cells were pelleted by centrifugation and plated on 7H10 Middlebrook agar, supplemented with 10% (*v*/*v*) ADC, 0.5% (*v*/*v*) glycerol, 50 μg/mL kanamycin, and 50 μg/mL hygromycin B. Colonies appeared after 2–3 days’ incubation at 37 °C. A single colony was used to inoculate 100 mL of 7H9/ADC/Tween80/glycerol, supplemented with 50 μg/mL kanamycin and 50 μg/mL hygromycin B. Bacteria were cultured for 20–22 h at 37 °C with shaking. This overnight culture was used to inoculate 5 L (at 1.5%) of 7H9 Middlebrook broth supplemented with 10% (*v*/*v*) ADC, 0.05% (*v*/*v*) Tween-80, 0.5% (*v*/*v*) glycerol, kanamycin, and hygromycin B, both at 50 μg/mL. Cultures were grown with shaking at 37 °C until the OD_600_ reached 0.4–0.7 before inducing SufS expression by adding 1 mM Isopropyl β-D-1-thiogalactopyranoside (IPTG). Induction was maintained for 48 h at 37 °C before harvesting cells by centrifugation at 5000 rpm for 10 min. Pellets were stored at −80 °C. For SufS purification, cells were resuspended (mg/mL) in buffer A (50 mM Tris pH 7.5, 150 mM NaCl) containing protease inhibitors and lysed by sonication (15 cycles of 10 s at 40% amplitude) using a Vibra-Cell™ VCX 500 sonicator (Sonics & Materials, Inc., Newtown, CT, USA). Lysates were centrifuged (40,000 rpm, 4 °C, 90 min) to remove cell debris. The supernatant was loaded on a prepacked HisTrap Hp column containing Ni Sepharose equilibrated with 10 bed volumes of buffer B (50 mM Tris pH = 7.5, 100 mM NaCl). Bound protein was eluted with buffer B containing 500 mM imidazole. Imidazole was removed using a HiPrep™ 26/10 Desalting column (Cytiva). Desalted protein was then loaded on a size-exclusion chromatography HiLoad™ 16/600 Superdex 200 column (Cytiva) equilibrated with buffer B. The fractions containing pure SufS protein were pooled and concentrated using Amicon^®^ ULTRA centrifugal filter devices (30 kDa molecular mass cutoff). Absorbance was measured at 280 nm using a NanoDrop1000 spectrophotometer according to the user manual, and protein concentration was determined by applying the molar extinction coefficient (25,900 M^−1^ cm^−1^). Pyridoxal phosphate bound to SufS was quantified by measuring absorbance at 415 nm (extinction coefficient: 6400 M^−1^cm^−1^).

*E. coli* Arctic express competent cells (Novagen) were transformed with pET15b-sufU plasmid by heat shock (30 s at 42 °C). Transformed bacteria were selected on LB-agar plates containing ampicillin (100 µg/mL) and gentamicin (20 µg/mL). Single colonies were used to inoculate 100 mL of LB with the same antibiotic concentration as the solid medium and incubated overnight at 37 °C. The overnight culture was used to inoculate 12 L of LB medium at 1.5%. Bacterial growth was allowed to proceed at 37 °C up to an OD_600_ = 0.5. Protein expression was then induced by adding 0.5 mM IPTG and continuing the culture for 16 h at 13 °C. Cells were harvested by centrifugation at 5000 rpm for 30 min and stored at −80 °C. For SufU purification, cells were resuspended in 100 mL buffer C (150 mM Tris pH = 8, 100 mM NaCl, 1% glycerol, 50 mM dithiothreitol (DTT), 30 mg lysozyme, cOmplete™ mini EDTA-free Protease Inhibitor Cocktail tablets), and lysed by three freeze/thaw cycles. Lysates were centrifuged (40,000 rpm, 4 °C, 90 min) to remove cell debris. The supernatant was loaded on a prepacked HisTrap Hp column containing Ni Sepharose equilibrated with 10 bed volumes of buffer D (150 mM Tris pH = 7.5, 100 mM NaCl). Bound protein was eluted with buffer D containing 500 mM imidazole. Imidazole was removed using a HiPrep™ 26/10 Desalting column (Cytiva). Desalted protein was then loaded on a size-exclusion chromatography HiLoad™ 16/600 Superdex 75 column (Cytiva) equilibrated with buffer D. The fractions containing SufU pure protein were pooled and concentrated using Amicon^®^ ULTRA centrifugal filter devices (10 kDa molecular mass cutoff). Absorbance was measured at 280 nm using a NanoDrop1000 spectrophotometer according to the user manual, and the protein concentration was determined by applying the molar extinction coefficient (13,075 M^−1^ cm^−1^). The SufU purification yield was 3 mg/L of culture.

### 2.4. SEC-MALLS-RI

An aliquot (20 µL) of the sample was loaded on an analytical Superdex S200 Increase size-exclusion chromatography (SEC) column (GE Healthcare, Tremblay-en-France) for SufS or on an analytical Superdex S75 Increase SEC column (GE Healthcare) for SufU. Both columns were pre-equilibrated with buffer A. SEC was performed at a flow-rate of 0.5 mL·min^−^^1^ with an in-line multi-angle laser light scattering (MALLS) spectrometer (DAWN HELEOS II, Wyatt Instruments). An in-line refractive index (RI) detector (Optirex, Wyatt Instruments) was used to follow the differential refractive index relative to the solvent. Masses were estimated by applying the Debye model using ASTRA software version 6 (Wyatt Instruments), with a theoretical dn/dc value of 0.185 mL·g^−^^1^.

### 2.5. Cysteine Desulfurase Activity

Cysteine-desulfurase activity was determined by quantifying sulfide based on methylene blue formation. Unless otherwise indicated, the reaction mixture (100 µL) containing 0.5 µM 6xHis-SufS was incubated with 2 mM DTT for 10 min, ±6xHis-SufU (apo, Zn- or Metal-containing SufU) in the presence of 500 µM L-cysteine in buffer E (50 mM MOPS, pH = 8) at 37 °C. The reaction was quenched by the addition of 12.5 µL DMPD (N,N-Dimethyl-p-phenylenediamine) (20 mM) and 10 µL FeCl_3_ (30 mM) to convert the released S^2−^ into methylene blue. The precipitated protein was separated by centrifuging samples at 10,000 rpm for 4 min. The amount of methylene blue was determined by measuring absorbance at 670 nm (Varian Cary^®^ 50 UV-Vis Spectrophotometer) and comparing it to a calibration curve produced with Na_2_S.

For assays in the presence of H_2_O_2_, SufS, SufE, and SufU were separately pre-incubated with 5 mM DTT for 30 min. DTT was then removed by passage on a MicroBiospin-6 column (Bio-Rad, France) under anaerobic conditions. Desulfurase reactions were initiated by adding 2 mM cysteine together with a range of H_2_O_2_ concentrations. Reactions were allowed to proceed for 30 min under anaerobic conditions before quenching by heating to 95 °C for 5 min. Samples were reduced by adding 1 mM DTT, releasing sulfide for measurement. Finally, DMPD and FeCl_3_ were added to develop methylene blue. After 30 min, data were analyzed using Simple-Read software of the Varian Cary^®^ 50 UV-Vis Spectrophotometer. The values for 100% S^2−^ production were 3.2 nmol/min/mg for *E. coli* SufS, 87.5 nmol/min/mg for *E. coli* SufS-SufE, 8 nmol/min/mg for *Mtb* SufS, and 139 nmol/min/mg for *Mtb* SufS-SufU.

### 2.6. SufU Affinity for Zinc

A 50 mM stock solution of N,N,N′,N′-tetrakis(2-pyridinylmethyl)-1,2-ethanediamine (TPEN) dissolved in 95% ethanol was prepared. Several dilutions of TPEN (0 mM, 0.5 mM, 1 mM, 2.5 mM, 5 mM, 10 mM) were then prepared in buffer D. As-isolated Zn-SufU (1.46 mM), containing zinc, was incubated with the different TPEN concentrations (final volume = 100 µL) for 2 h at room temperature. Solutions were then dialyzed three times (2 h, 4 °C) in 2 L of buffer F (50 mM Tris, pH 8). The cysteine-desulfurase activity of SufS was finally measured in the presence of SufU from the different samples. The zinc-affinity of SufU was determined using the equation: $Ka\;SufUzn=Ka\;TPEN\frac{\left[SufUzn\right]\left[TPEN\right]}{\left[SufUapo\right]\left[TPENzn\right]}$. The SufU_zn_ concentration in each sample was determined from a calibration curve of SufS cysteine-desulfurase activity in the presence of a range of sub-saturating SufU_zn_ concentrations. [Apo-SufU] = initial SufU concentration (as-isolated with 1.1 zinc)—[SufU_Zn_] and [TPEN_zinc_] = [Apo-SufU]. The amount of zinc in each sample was also determined by ICP-AES. The zinc content and the SufU-related desulfurase-stimulating activity of SufS with 0 mM TPEN were taken as 100%.

### 2.7. Apo-SufU Preparation

Apo-SufU was prepared by incubating 200 µM of as-purified 6xHis Zn-SufU (containing 1.1 zinc/monomer) (400 µL) with 30 mM of the metal-chelating agent diethylenetriaminepentaacetic acid (DTPA) dissolved in buffer G (1 M Tris pH = 8) for 2 h at room temperature. The solution was then submitted to three dialysis cycles (4 °C, 2 h) in 2 L of buffer H (150 mM Tris pH = 8, 100 mM NaCl, 10% glycerol), and the protein was finally loaded onto a HiLoad™ 16/600 Superdex 75 SEC column equilibrated with buffer D to remove remaining traces of DTPA.

### 2.8. Reconstitution of SufU with Zinc

The nickel ion was removed from nickel nitrilotriacetic acid (Ni-NTA) resin by washing with 10 bed volumes of 3% (*v*/*v*) HCl until the resin turned completely white. The column was then washed with 10 bed volumes of distilled water before loading with one bed volume of 0.2 M ZnCl_2_ (pH < 5.5). The column was washed once again with 10 bed volumes of distilled water to remove excess unbound ions and then equilibrated with 5 bed volumes of buffer I (50 mM Tris-HCl pH = 7.5, 600 mM NaCl) to remove any remaining unbound metal ions. The column was then re-equilibrated with 5 bed volumes of protein buffer J (150 mM Tris-HCl pH 8, 100 mM NaCl). The suspension of 6xHis-apo-SufU was loaded onto the column and eluted with buffer J containing 500 mM Imidazole. Imidazole was subsequently removed using a desalting column equilibrated with buffer J.

### 2.9. Reconstitution of SufU with Divalent Metal Ions

6xHis-Apo-SufU (100 µM) was incubated with 0.5 mM of metal solution (Ferric chloride, ammonium iron (II) sulfate hexahydrate, Zinc chloride, Cobalt (II) chloride hexahydrate (Acros Organics, Cole-Parmer, France), copper (II) chloride dehydrate, manganese chloride tetrahydrate, Nickel (II) sulfate hexahydrate (Acros Organics), or nickel(II) sulfate hexahydrate (Acros Organics)) and 0.5 mM EDTA in buffer E (final volume = 50 µL) for 3 h at room temperature. Samples were then loaded onto a NAP-10 column equilibrated with buffer E to remove excess metal.

### 2.10. ICP-AES

Metal concentrations were determined by inductively coupled plasma atomic emission spectroscopy (ICP-AES) (Shimadzu ICP 9000 with mini plasma torch) in axial reading mode. SufU was mineralized by incubation in 65% (*v*/*v*) HNO_3_ for 16 h at 60 °C. The volume was brought up to 6 mL with pure water. Standard solutions of Ni, Co, Mo, Mg, and Fe for atomic absorption spectroscopy (Sigma-Aldrich, Saint-Quentin-Fallavier, France) were used for quantification (calibration curve between 1.9 and 5000 μg L^−^^1^ in 10% HNO_3_ (Fluka, Illkirch-Graffenstaden, France).

### 2.11. 6xHis-Tag Cleavage from 6xHis-SufS and 6xHis-SufU

*6xHis-SufS.* A TEV (Tobacco Etch Virus) cleavage site was inserted between the coding sequences of the His_6_ tag and the N-terminus of the *Mtb* SufS. To cleave the 6xHis-tag, 6xHis-SufS was incubated at room temperature for 16 h with 6xHis-TEV (homemade enzyme) (60:1) and 0.5 mM EDTA. The mix was then passed through an affinity column (Ni-NTA) and washed with buffer K (50 mM Tris pH: 8, 150 mM NaCl). Cleaved SufS was present in the flow-through (yield: 61%). The 6xHis-TEV and uncleaved 6xHis-SufS were eluted by adding 500 mM imidazole to buffer K.

*6xHis-SufU.* A thrombin cleavage site was inserted between the coding sequences of the His_6_ tag and the N-terminus of the *Mtb* SufU. To cleave the 6xHis-tag, 6xHis-SufU was incubated at 4 °C for 16 h with thrombin (Cytiva) (1 mg of protein: 1 unit of thrombin). The mix was then loaded on an affinity column (Ni-NTA) equilibrated with buffer J. The cleaved SufU was present in the flow-through (yield: 68%). Uncleaved 6xHis-SufU was eluted by adding 500 mM imidazole to buffer J.

### 2.12. Fe-S Cluster Reconstitution in SufU

All steps were performed under anaerobic conditions inside a Jacomex glovebox (<1 ppm oxygen). SufU (Apo-form or Zinc-bound form, 56 µM) was pretreated anaerobically with 5 mM DTT in buffer J before incubation with 0.5 µM 6xHis-SufS and 500 µM L-cysteine for 30 min. Then, 50 mM of ammonium iron (II) sulfate hexahydrate was added, and UV-visible spectra (250–800 nm) were continuously recorded on a UVIKON XL spectrophotometer to monitor cluster formation. Unbound iron and sulfide were removed by passage on a NAP-25 column (Cytiva). The 6xHis-SufS was removed by loading the mixture onto a nickel nitrilotriacetic acid (Ni-NTA) column equilibrated with buffer J, and reconstituted SufU was recovered in the flow-through (the 6xHis-tag was cleaved before reconstitution). The amount of Fe and S content was determined using the Fish and Beinert methods, respectively [31,32].

### 2.13. UV-Visible Absorption Spectroscopy

UV-visible spectra were recorded with a Uvikon XL (Bio-Tek instrument, France) spectrophotometer connected to the glove box by optical fibers.

### 2.14. Circular Dichroism Analysis

The secondary structure of each protein was determined by Circular dichroism (CD) (JASCO J-815 CD spectrometer). All samples were prepared at 5 µM in 6 mM HEPES, pH 7.5, 10 mM NaCl and were analyzed in a 1-mm path-length quartz cell. Scans were collected in the far-UV region from 190 to 250 nm at 1 nm intervals and a scan-rate of 50 nm/min. Each CD spectrum corresponds to the average of 10 accumulated scans after baseline correction by subtracting the blank.

### 2.15. X-ray Crystallography

Solutions of purified *Mtb* SufS and *Mtb* SufU, both at a concentration of 2.4 mM in a 10 mM MOPS buffer at pH 6.7, were mixed in a stoichiometric ratio to produce a 2.4 mM *Mtb* SufS-SufU solution. *Mtb* SufS-SufU complex crystals were grown using the hanging drop vapor diffusion technique at 293 K. The crystallization drops, which consisted of a mixture of 1 μL of protein-complex solution with an equal amount of crystallization solution (0.4 M KNO_3_, 16% PEG 3350, 0.1M MOPS at pH 6.7), were equilibrated against 1 mL of crystallization solution in the reservoir. Yellowish elongated crystals (>100 μm long) appeared within a few days. Crystals were harvested and cryoprotected in the mother liquor complemented with 30% glycerol before flash-freezing in liquid nitrogen. A series of *Mtb* SufS-SufU crystals were screened on the micro-diffractometer of the ID30B beamline (ESRF, Grenoble, France) [33] at 100 K using a Flex-HCD sample changer [34]. After selecting the best crystal, a full diffraction dataset was collected at a wavelength of 0.9763 Å using an Eiger 9M detector (Dectris, Baden, Switzerland). Experimental diffraction data were processed (integrated, scaled, merged, and converted into the CCP4 format) using the XDS program suite [35]. The first phases were obtained by molecular replacement using the Phaser program [36], taking the *Bacillus subtilis* SufS-SufU structure as the initial model [24] (PDB deposition 5XT5). On this basis, a structural model was automatically built by the ARP/wARP program [37] using the real sequence of the SufS-SufU complex from *Mycobacterium tuberculosis*. Finally, this model was rebuilt and refined using Coot [38] and Refmac [36], respectively. The data collection and refinement statistics are reported in Table S1. The structure of *Mtb* SufS-SufU has been deposited in the protein data bank with the entry 8ODQ.

## 3. Results

### 3.1. Purification of Mtb Rv1464 and Rv1465

Cysteine desulfurases are essential enzymes for the mobilization of sulfur in biomolecules [39,40]. These 5′-pyridoxal-phosphate (PLP)-dependent enzymes use L-cysteine as the source of sulfur. The overall mechanism proposed for cysteine desulfurases involves two half-reactions: a desulfurase reaction, during which L-cysteine is converted to alanine and a persulfide species on the enzyme, and a transpersulfuration reaction, during which sulfur from the persulfide is transferred to a sulfur acceptor molecule [21]. Based on sequence alignment, these enzymes have been categorized into two types [39,41]. Type-specific signature motifs (in their tertiary structural contexts) provide a rationale for catalytic differences and for specific interactions with sulfur acceptors. Type I cysteine desulfurases, with IscS and NifS as prototypes, are characterized by a catalytic cysteine residue located in a conserved flexible loop, located at 23 Å from the PLP [42]. This structural feature allows the persulfide sulfur to move away from the PLP active site, thus favoring the transpersulfurase reaction with various sulfur acceptors [43,44,45,46]. Type II cysteine desulfurases, with SufS and CsdA as prototypes, are characterized by a catalytic cysteine located in a smaller, more rigid loop located ~7 Å from the PLP cofactor [47,48]. An additional feature of Type II enzymes is the 19-residue insertion before the catalytic cysteine residue. This sequence forms a β-hairpin motif implicated in the proposed “half-sites” regulatory mechanism of these enzymes [49]. Other hallmarks of type II enzymes include the presence of a glycine-rich region and a proline residue located just before and after the β-hairpin motif, respectively. In combination with the β-hairpin-loop, they form a β-latch motif that makes the catalytic cysteine of SufS accessible to its partners and thus allows the transpersulfuration reaction to occur [50,51].

**Figure 2:**
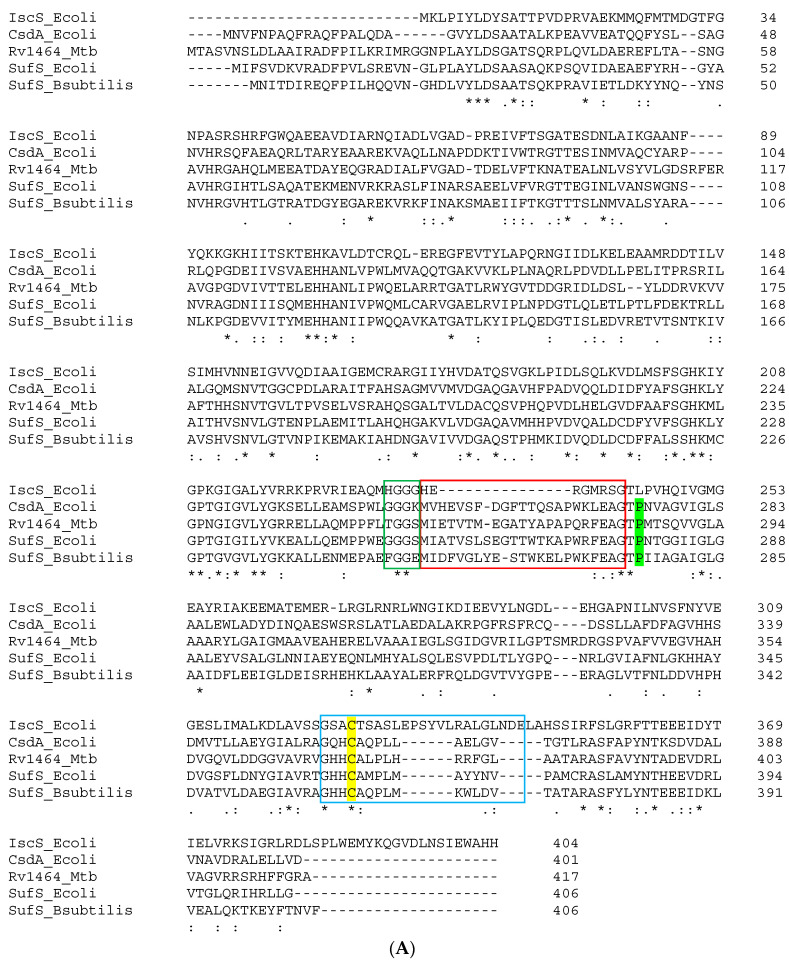

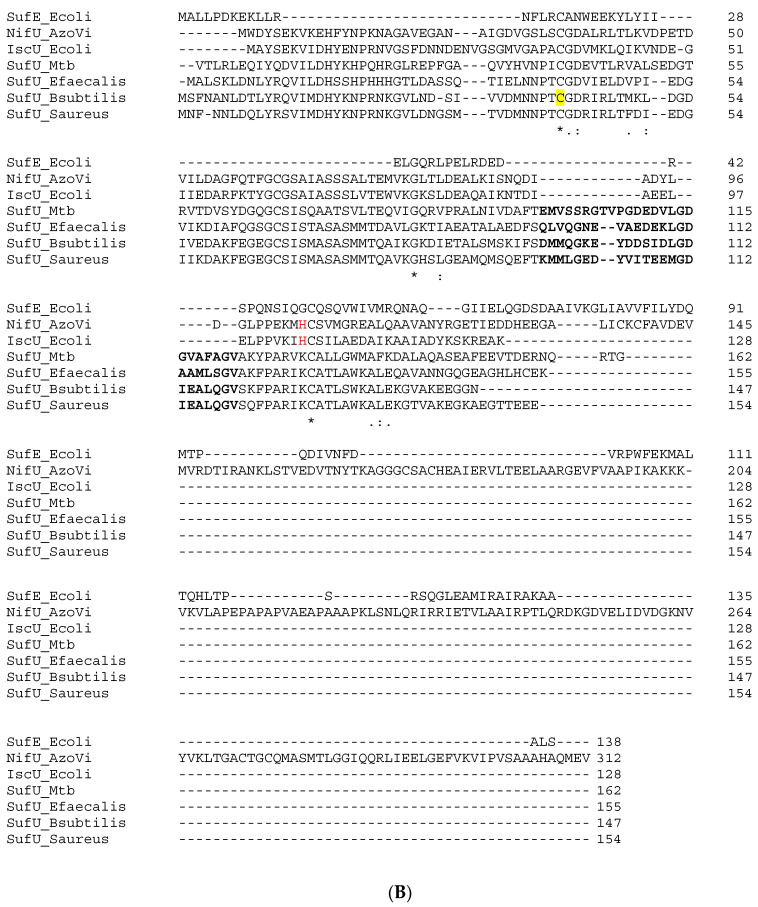
(**A**) Sequence alignment of Rv1464 with IscS (*E. coli*), CsdA (*E. coli*), SufS (*E. coli* and *B. subtilis*); green box: glycine-rich region; red box: β-hairpin; blue box: catalytic loop containing the conserved cysteine; yellow highlight: catalytic cysteine; green highlight: conserved proline characteristic of type II cysteine desulfurases; (**B**) Sequence of Rv1465 aligned with SufE and IscU (*E. coli*), NifU (*A. vinelandii*) and SufU (*S. aureus*, *E. faecalis*, and *B. subtilis*). Yellow highlight: sulfur acceptor cysteine (*B. subtilis*); red: conserved histidine residue characteristic of NifU/IscU proteins; bold: sequence insertion in SufU proteins.

Alignment of the amino acid sequence of the protein encoded by Rv1464 with other cysteine desulfurases from *E. coli* and *B. subtilis* (IscS, CsdA, SufS) confirmed that Rv1464 is probably a type II cysteine desulfurase (Figure 2A). Indeed, it shares all the features mentioned for type II enzymes (Figure 2A). Rv1464 displays 43% identity with *B. subtilis* SufS, 45% identity with *E. coli* SufS, and 37% identity with *E. coli* CsdA (but only 30% identity with *E. coli* IscS).

Rv1465 is annotated by the mycobrowser site as a nifU-type protein, scaffold of the nif operon in nitrogen-fixating bacteria [52]. Alignment of the amino acid sequence of the protein encoded by Rv1465 with other proteins involved in Fe-S assembly, such as the scaffolds NifU and IscU, and sulfurtransferases SufE and SufU, revealed Rv1465 to be more likely a SufU protein. Indeed, similar to *B. subtilis*, *S. aureus*, and *E. faecalis* SufU (but unlike *E. coli* SufE), Rv1465 contains a 21–23 amino acid sequence inserted between the second and third cysteine residues. This sequence is absent in IscU/NifU. In addition, a conserved lysine residue is present in Rv1465, occupying the position of the essential histidine (one ligand of the Fe-S cluster) preceding the third conserved cysteine in IscU/NifU (Figure 2B). Mtb SufU shares 36% sequence identity with *B. subtilis* SufU, 33% identity with *S. aureus* and *E. faecalis* SufU, but just 16% sequence identity with *E. coli* SufE.

Taken together, this analysis of amino acid sequences suggests that Rv1464 is a type II SufS cysteine desulfurase, whereas Rv1465 is a SufU protein.

**Figure 3:**
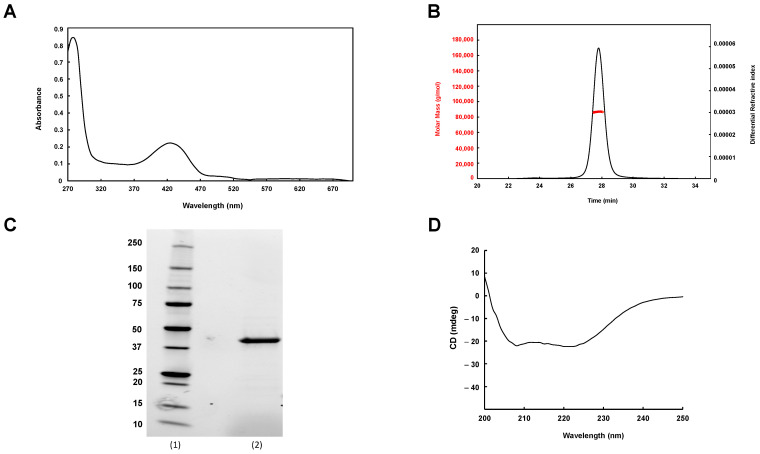
Characterization of *Mtb* SufS. (**A**) UV-Vis absorption spectrum of *Mtb* SufS showing PLP absorption at 420 nm (1 µM SufS contains 1.01 µM PLP). (**B**) Characterization of *Mtb* SufS by Size-Exclusion Chromatography combined with Multi-Angle Light Scattering (SEC-MALLS). (**C**) SDS-PAGE profile of purified *Mtb* SufS. Lane (1): molecular weight markers (in kDa), lane (2): SufS after purification on a Ni-NTA column, Superdex-200, and 6xHis tag cleavage. (**D**) Far-UV CD spectrum of purified SufS (5 μM) in 6 mM HEPES, pH 7.5, 10 mM NaCl at 25 °C.

To confirm that Rv1464 and Rv1465 correspond, respectively, to SufS and SufU proteins, both were expressed, purified, and characterized. *Mtb* SufS was overexpressed in *M. smegmatis* cells (strain mc^2^4517) using the pYUB28b vector. After two-step purification on a Ni-NTA column followed by gel filtration, its molecular mass was determined by SEC-MALLS. A main peak was observed at 87.017 kDa, corresponding to a dimer. As expected for a cysteine-desulfurase protein, Rv1464 had a yellowish color, in agreement with the presence of a protein-bound pyridoxal phosphate (PLP) cofactor (1.01 PLP/monomer). The *Mtb* SufS protein was obtained with a good yield—20 mg/L of culture. CD spectroscopy in the far-UV range revealed a typical alpha helix protein signature, with a positive peak at 195 nm and two negative peaks at 205 and 225 nm (Figure 3).

**Figure 4:**
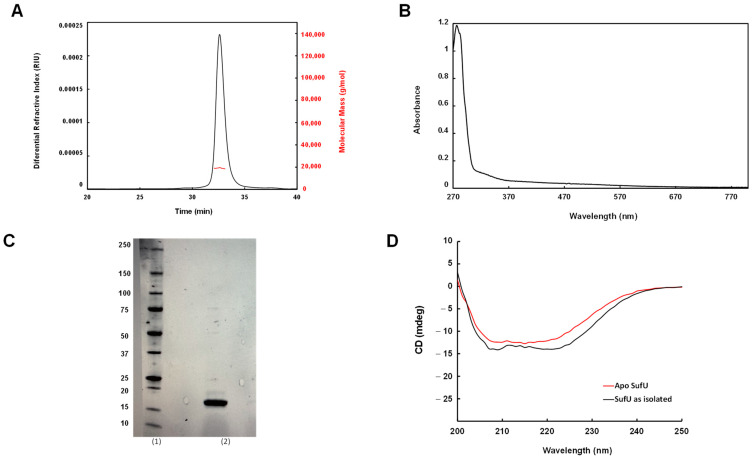
Characterization of WT *Mtb* SufU. (**A**) Size-Exclusion Chromatography combined with Multi-Angle Light Scattering (SEC-MALLS). (**B**) UV-Vis absorption spectrum of purified *Mtb* SufU (1.5 mg/mL); (**C**) SDS-PAGE profile of purified *Mtb* SufU. Lane (1): molecular weight markers (in kDa) and lane (2): SufU after purification on Ni-NTA column, Superdex-75 gel filtration, and 6xHis tag cleavage. (**D**) Far-UV CD spectrum of as-isolated SufU and apo-SufU (5 µM) in 6 mM HEPES, pH 7.5, 10 mM NaCl at 25 °C.

Attempts to express SufU in M. smegmatis failed. Consequently, the protein was expressed in *E. coli* Artic Express DE3 cells and purified under aerobic conditions using Ni-NTA and gel filtration chromatography. After cleavage of the 6xHis tag, the recombinant protein eluted from a Superdex-75 gel filtration column was present in two peaks. SEC-MALLS analysis revealed a major peak corresponding to a size of 17 kDa and a minor peak corresponding to SufU aggregates (Figure 4). Based on these observations, in solution, SufU mainly exists as a monomer. Analysis of the gel filtration fractions by SDS-PAGE revealed a unique, clean band, indicating that the protein was more than 95% pure. The purification yield was 3 mg/L of culture. CD spectroscopy in the far-UV range revealed a typical alpha helix protein signature, with a positive peak at 195 nm and two negative peaks at 205 and 220 nm (Figure 4). The UV-visible absorbance spectrum of purified recombinant Rv1464 presented no characteristic absorption bands indicative of a metal-bound protein (Figure 4). However, ICP-AES analysis of purified *Mtb* SufU (Table 1) revealed the presence of significant levels of zinc (1.1 ± 0.2 zinc/monomer), a low Ni content (0.1/monomer), but, interestingly, no Fe.

### 3.2. Zn Is Tightly Bound to Mtb SufU

**Figure 5:**
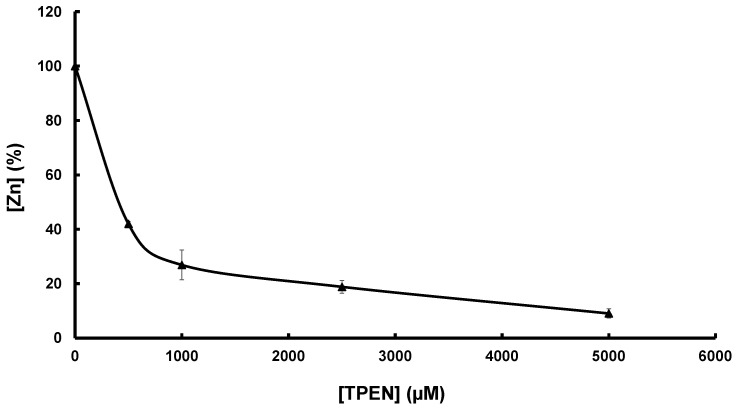
Affinity of SufU for zinc. SufU (146 μM) was incubated with increasing concentrations of TPEN (0–5 mM) for 3 h and then dialyzed. The amount of zinc-bound SufU was determined by ICP-AES (▲). [Zn] 100% corresponds to 1.1 ± 0.2 Zn per SufU monomer.

Mtb SufU is a zinc-containing protein. However, the concentration of this metal in LB medium, estimated to be approximately 10 μM [34], could lead to adventitious metal incorporation during protein expression. To verify whether this was the case, the binding affinity of Mtb SufU for zinc was determined by a titration experiment with the zinc chelator TPEN [53,54] (Figure 5). As-isolated SufU (1.1 zinc/protein) was incubated with various concentrations of TPEN and then dialyzed. The remaining zinc bound to the protein, determined by ICP-AES, was plotted as a function of the TPEN concentration (Figure 5). Using the known binding affinity of TPEN for zinc (10^16^ M^−1^), the Ka of SufU for zinc was calculated as 4.23 10^16^ M^−1^. This value corresponds to a high affinity constant for zinc-dependent enzymes [55].

### 3.3. Mtb SufS Displays a Cysteine Desulfurase Activity and a Half-Site Reactivity

**Figure 6:**
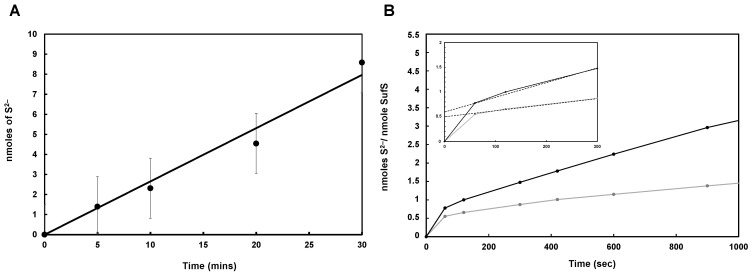
SufS activity. (**A**) Cysteine-desulfurase activity of *Mtb* SufS (1 nmol) in the presence of L-Cysteine (500 µM). (**B**) Enzymatic activity of SufS (3.5 nmol) in the presence of L-cysteine (500 µM) over 900 s in the absence (●) and presence (●) of 1 mM DTT. Inset: zoom of the 0–300 s period. The dotted straight lines correspond to the extrapolation of the experimental line to the *y*-axis.

Cysteine desulfurases mobilize sulfur from cysteine, forming alanine and a persulfide species on a conserved cysteine residue (Cys 364 in *E. coli*; Cys361 in *B. subtilis*). The cysteine-desulfurase activity of *Mtb* SufS was measured using L-cysteine as substrate. The enzymatic activity was determined by quantifying the amount of sulfide released from L-cysteine 30 min after the start of the reaction, using the formation of methylene blue as a read-out. Similar to SufS from *B. subtilis* and *E. coli* [14,15,56], *Mtb* SufS turned at a very modest rate (Figure 6A). The low specific activity (specific activity = 5 nmol/min/mg) is due to the limited reactivity of the persulfurated enzyme since the cleavage of the persulfide bond on SufS enzymes dictates the overall reaction rate. We plotted the number of nmol of S^2−^/nmol of *Mtb* SufS as a function of time (Figure 6B). The number of active sites was estimated by extrapolation of the steady-state formation of sulfide over time. The amplitude of the y-intercept, which indicates the number of the active site(s), was calculated to be 0.55 nmol of sulfide/nmol of SufS. This result suggests that, in the first turnover, just over half (55%) of the enzyme is active. Purified *Mtb* SufS contains 1.0 ± 0.5 PLP/SufS, indicating that the substoichiometry of active sites on SufS is not due to low cofactor occupancy. Altogether, these data strongly suggest that *Mtb* SufS, similar to other SufS enzymes, has a half-site reactivity [13,21].

Interestingly, exposure to 1 mM DTT increased the amplitude of the y-intercept (0.72 nmol of sulfide/nmol of SufS) (Figure 6B), suggesting that the purified SufS enzyme contained some persulfurated species (SufS-SH). The presence of these species was confirmed biochemically by measuring the sulfur content in the as-isolated SufS (0.7 sulfur/ SufS monomer) and by native mass spectrometry analysis.

### 3.4. Mtb Zn-SufU Is a Substrate of SufS

**Figure 7:**
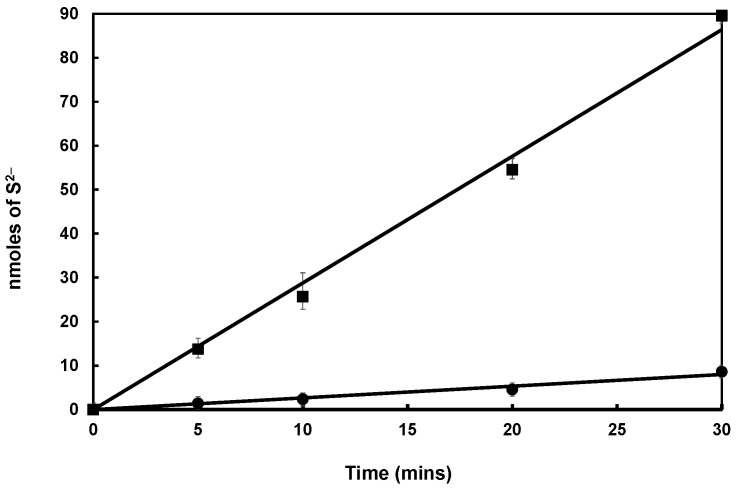
Cysteine-desulfurase activity of SufS. Activity of 1 nmol *Mtb* SufS in the presence (black square) or absence (black circle) of 1 nmol *Mtb* SufU and 500 µM L-Cysteine.

**Figure 8:**
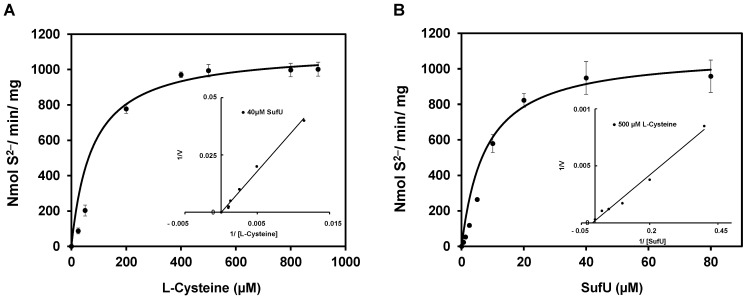
(**A**) Cysteine-desulfurase activity of *Mtb* SufS (0.5 µM) in the presence of L-cysteine (500 µM), DTT (2 mM), and increasing concentrations of SufU (0–80 µM). (**B**) Cysteine-desulfurase activity of *Mtb* SufS (0.5 µM) in the presence of SufU (40 µM), DTT (2 mM), and increasing concentrations of L-cysteine (0–1 mM). The lines are the best fits to the Michaelis-Menten equation obtained using Sigmaplot.

The cysteine-desulfurase activity of SufS was increased by one order of magnitude in the presence of stoichiometric amounts of Zn-SufU (specific activity = 59 nmol/min/mg) (Figure 7). This effect suggests that Zn-SufU is an active participant in the catalytic mechanism of SufS. We next measured SufS activity in the presence of increasing Zn-SufU concentrations (Figure 8A). The saturation curve exhibited a hyperbolic response and a straight line in the double-reciprocal Lineweaver-Burk plot. This profile corroborates the hypothesis that SufU acts as a substrate of SufS. The same experiment performed with increasing L-cysteine concentrations confirmed that L-cysteine is also a substrate (Figure 8B). The specific activity of *Mtb* SufS obtained with 80 equiv. of Zn-SufU and 800 equiv. of L-cysteine was around 900 nmol/min/mg of SufS. This activity is within the range of that reported for type II cysteine desulfurases, such as SufS from *E. coli* (877 U/mg) and *B. subtilis* (1100 U/mg) [13,21].

As SufE from *E. coli* is a substrate of *E. coli* SufS [13], we tested whether *E. coli* SufE could activate Mtb SufS in the same way as Mtb SufU. We found that SufE could not activate Mtb SufS, even at a SufS:SufE ratio of 1:80 (Figure S1). This result strongly suggests that Mtb SufS displays substrate specificity.

### 3.5. Mechanism of SufS Activity

**Figure 9:**
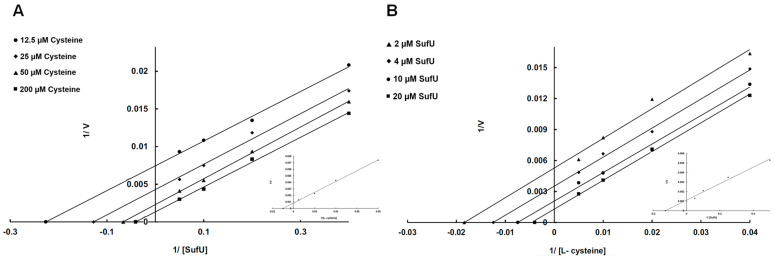
Double-reciprocal plots of sulfide formation. Steady-state rates of sulfide formation were determined with varying concentrations of SufU (2–20 µM) in the presence of 12.5 µM (●), 25 µM (◆), 50 µM (▲), and 200 μM (■) L-cysteine (**A**), or variable concentrations of L-cysteine (12.5–200 µM) against several fixed concentrations (2 µM (▲), 4 µM (◆), 10 µM (●), and 20 µM (■)) of SufU (**B**). The lines in the plots correspond to linear fits generated by applying the Lineweaver-Burk equation. The inset in panel A shows the replot of the y-intercept [V_max,app_ = 1/V_max_(1 + K_m_Cys)/[Cys]] vs. 1/[Cys] fitted to a linear equation. The inset in panel B shows the replot of the y-intercept [V_max,app_ = 1/V_max_(1 + K_m_SufU)/[SufU]] vs. 1/[SufU] fitted to a linear equation. The x-intercepts from these two fits give −1/K_m_ Cys and −1/K_m_ SufU, respectively. The y-intercepts of the insets give the corresponding V_max_ values. All kinetic constants are listed in Table 2.

SufS activity in the presence of SufU suggests that SufU is a sulfurtransferase, taking the persulfide sulfur from SufS to regenerate the SufS catalytic cysteine. The transfer of sulfur from L-cysteine to SufU was characterized by a two-substrate kinetic analysis, in which the concentration of one substrate was kept constant while the second one varied (Figure 9). Double-reciprocal plots generated with a range of SufU or L-Cysteine concentrations consisted of parallel lines, which are typical of a ping-pong mechanism. A replot of the double-reciprocal plots of apparent V_max_ values versus SufU concentration (1/V_max,app_ × 1/[SufU]) and Cys concentration (1/V_max,app_ × 1/[Cys]) (Figure 9, inset) returned the SufS kinetic parameters K_m_ and V_max_ (Table 2). Based on a ping-pong mechanism, the model for the cysteine-SufU sulfurtransferase reaction involves the formation of a persulfide SufS enzyme and the release of alanine before binding the SufU protein. According to this mechanism, SufU must interact with SufS in solution during the reaction. This interaction was demonstrated using a batch purification technique and passage on a Ni-NTA resin (Figure S2).

### 3.6. Zinc Bound to SufU Is Required for SufS Activity

**Figure 10:**
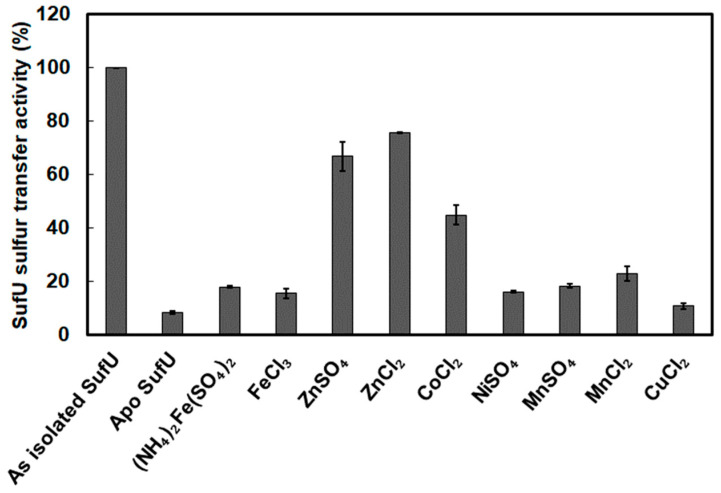
Sulfurtransferase activity (%) of SufU previously incubated with distinct divalent metals. SufU (10 µM) was mixed with SufS (5 µM) and cysteine (500 µM). Activity with as isolated SufU = 68 nmol/min/mg (100%). As isolated SufU contains 1.1 Zn/monomer.

We investigated whether zinc is an important functional element for sulfur mobilization by SufS and SufU, as reported for *B. subtilis* [22]. To do so, we measured the catalytic competence of apo-SufU as substrate. Apo-SufU was prepared using DTPA treatment, and its ability to act as a SufS substrate was investigated by measuring sulfide production, as described above. The apo-SufU (0.1 Zn/monomer determined by ICP-AES) weakly activated SufS (10%) (Figure 10). When apo-SufU was reconstituted with Zn (1.3 Zn/ monomer determined by ICP-AES, Figure S3), it fully activated SufS (96% activation relative to the control) (Figure S3). These results demonstrate that zinc is essential for SufU sulfurtransferase activity.

The importance of the zinc ion in the sulfurtransferase activity was also demonstrated by measuring sulfur production in the presence of SufS and TPEN-treated Zn-SufU (following dialysis) (Figure S4). The greater the concentration of TPEN added to SufU, the lower the catalytic activity. Therefore, the more apo-SufU present, the lower the activity. Interestingly, the sulfurtransferase activity measured correlated directly with the amount of zinc remaining associated with SufU after dialysis, as quantified by ICP-AES (Figure 5).

To determine whether zinc is specifically required for SufU activity, apo-SufU was incubated with several alternative divalent metallic ions (Fe^2+^, Fe^3+^, Ni^2+^, Mn^2+^, Co^2+^, and Cu^2+^), and sulfur production was monitored using Zn^2+^ as a control. With Zn^2+^, SufU regained 80% of its activity in the presence of SufS (Figure 10). With Fe^2+^, Fe^3+^, Ni^2+^, Mn^2+^, and Cu^2+^, there was no recovery of SufU sulfurtransferase activity, indicating that binding of metals other than Zn to SufU is either not tight enough or does not produce an active site that contributes to the sulfur transfer reaction. Interestingly, incubation with Co^2+^ did promote SufS activation (50%), as often observed with known zinc-binding proteins [57,58,59].

Altogether, these results demonstrate that the activity of *Mtb* SufU is dependent on the zinc bound to its polypeptide chain.

### 3.7. SufU as a Sulfur Acceptor Protein

The cysteine-SufU sulfurtransferase activity of SufS requires a thiol group on SufU to affect the transfer of a sulfur atom from SufS persulfide to SufU. SufU should promote the nucleophilic attack on the terminal persulfide thiol enzyme intermediate, controlling the overall reaction rate. Mtb SufU contains three cysteine residues (Cys31, Cys40, and Cys67) (Figure 2B). Assuming that Mtb SufS-SufU functions such as *B. subtilis* SufS-SufU, then Cys40 in Mtb SufU should be the residue accepting the persulfide from SufS. This hypothesis was confirmed by the crystal structure of the Mtb Zn-SufU-SufS complex, solved at 1.65-Å resolution.

**Figure 11:**
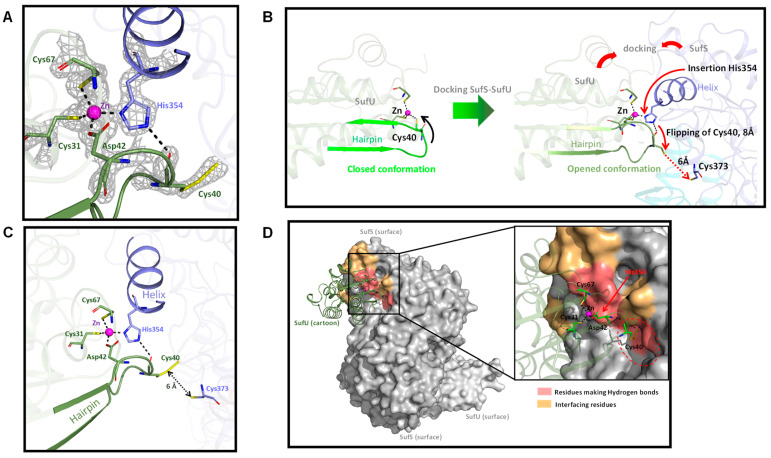
*Mtb* SufU-SufS interface interactions. (**A**) Structural details of the zinc coordination involving Cys31, Asp42, and Cys67 from SufU (in green) and His354 from SufS (in blue); (**B**) Details showing the insertion of His354 (SufS) between the Zn atom and Cys40-SSH (SufU) causing the hairpin bearing Cys40-SSH to move toward Cys373 (SufS); (**C**) SufU-SSH and SufS-SH; (**D**) Contacts between SufS (in surface representation) and SufU (in cartoon representation). Hydrogen bonds located close to the Zn atom and Cys40-SSH are represented in pink; other contacts (electrostatic and hydrophobic interactions) are located in a second shell, colored orange.

In this structure, the SufS-SufU complex is in the (SufS)_2_-(SufU)_2_ state (Figure S5). The Zn ion is tetracoordinated by three conserved residues in Mtb SufU (Cys31, Cys67, and Asp42) and the conserved His354 in Mtb SufS (Figure 11A). This zinc coordination sphere is identical to that previously reported in *B. subtilis* [24]. His354 is well conserved among SufS in bacteria where the SUF system includes a SufU protein, such as *B. subtilis*, *S. aureus*, Myxococcus xantus, and Enterococcus faecalis (Figure S6) [22,60,61,62].

The structure of the complex shows that His354, which is located at the extremity of an alpha helix and points out of the surface of SufS, is inserted between the Zn ion and Cys40 in SufU (Figure 11A). The imidazole side-chain forms a coordination bond with the Zn ion on one side and a hydrogen bond with the carbonyl oxygen of Cys40 on the other. To disclose the conformational changes that SufU undergoes upon docking to SufS, the structure of the Mtb SufU unit (as it is in complex) extracted from Mtb SufS-SufU was aligned on the structure of the isolated *B. subtilis* SufU, deposited in the PDB (6JZV). The root mean square deviation (RMSD) between the two structures for the Cα atoms, of 1.26 Å, indicates a high structural similarity. However, the superimposition revealed the hairpin loop Asn37-Gly41 of SufU (including Cys40) to undergo a major reorientation when docking to SufS. Indeed, in SufU alone in the solvent, residues Asp42, Cys31, Cys40, and Cys67 all coordinate the zinc atom, and the hairpin loop is in a closed conformation, de facto hiding the Cys40Sγ atom from the solvent. In contrast, when SufU and SufS interact, a sequential molecular mechanism ultimately leads to the accurate positioning of the SufU Cys40 side-chain in a cavity in SufS near Cys373. This is most likely a key conformational modification during the catalytic process (Figure 11B). This mechanism, which has never previously been described, can be schematically dissected as follows: first, SufU docks onto SufS; second, the zinc atom swaps its ligand from Cys40 to His354; third, the released SufU hairpin loop undergoes a major reorientation movement from the closed to the open-conformation; fourth, His354 forms a new hydrogen bond with the Cys40 carbonyl oxygen to stabilize the hairpin in the open state and create a protrusion inside SufS; finally, the Cys40 side-chain flips into the dedicated cavity of SufS to accurately position Cys40Sγ, via hydrogen bonding, close to Cys373Sγ (6 Å). We estimated the total displacement of Cys40Sγ, from Zn toward Cys373Sγ, to be ~14 Å. In this position, Cys40 can accept sulfur atom(s) from SufS. In agreement, in the Mtb SufS-SufU structure, the electron density map at Cys40 revealed an extra density that could be modeled as Cys40 persulfide (Cys40-SSH) (Figure 11A,C). Taken together, these structural data identify Cys40 in SufU as the sulfur acceptor site during the sulfurtransferase reaction with SufS.

The structural study of Mtb SufS-SufU revealed the essential role of the His354 residue in mediating the formation of the SufS-SufU complex via zinc coordination. Moreover, this His354 was found to stabilize Cys40, and thus the hairpin loop in its open state, by forming a hydrogen bond. This configuration leaves Cys40 accessible for sulfur transfer, suggesting that this amino acid plays an essential role in the sulfurtransferase activity of the complex (Figure 11B). We confirmed this hypothesis biochemically by measuring sulfur production using the *E. coli* SufS protein, which lacks this histidine residue, in the presence of Mtb SufU and L-cysteine. A very low sulfur yield was obtained (4 nmol/min/mg with SufS:SufU at a 1:20 ratio). This yield was the same as that produced by *E. coli* SufS alone (Figure S7), strongly suggesting that no sulfurtransferase reaction occurred between *E. coli* SufS and Mtb SufU. This lack of sulfurtransferase activity is likely due to the inability of *E. coli* SufS to coordinate the SufU zinc ion and, therefore, to make the Cys40 residue accessible for sulfur transfer. Thus, the absence of His354 in the *E. coli* SufS sequence, replaced by a poor Zn ligand (a tyrosine), caused a major functional deficit in the *E. coli* SufS/Mtb SufU system for sulfur production. Taken together, these results reveal how this conserved histidine residue contributes to SufS-SufU complex formation and its functional role.

Analysis of the interface contacts between SufS and SufU provides complementary information on Mtb SufS-SufU (Figure 11D). The first contact shell is localized around the functional residues (metallic site ligands and Cys40) and relies on a hydrogen bond network. The remaining hydrophobic and electrostatic contacts between SufS and SufU surround the zinc-binding site and constitute a second shell around the metal (Figure 11D and Table S2). The two protein surfaces dock in a very specific way, involving a few residues (14% of SufU residues and 5% of SufS residues) (contact area: 11% of SufU and 4% of SufS) (Table S2). The specificity of the residues at the interface between the two proteins is most likely responsible for the docking and very precise positioning of one protein relative to the other. Among the residues involved in interaction at the interface, some residues forming hydrogen bonds are strictly conserved (His352, His354, Asp355, and His371 in SufS; Tyr9, Cys40, Gly41, Ser68, and Arg128 in SufU). Similarly, Arg368 and Gly370 in SufS; Leu5, Asp42, Gly66, Cys67, and Cys131 in SufU, involved in other types of contact between SufS and SufU, are strictly conserved and thus are likely to be essential.

### 3.8. Mtb Zn-SufU Is Not an Intermediate in Fe-S Formation

**Figure 12:**
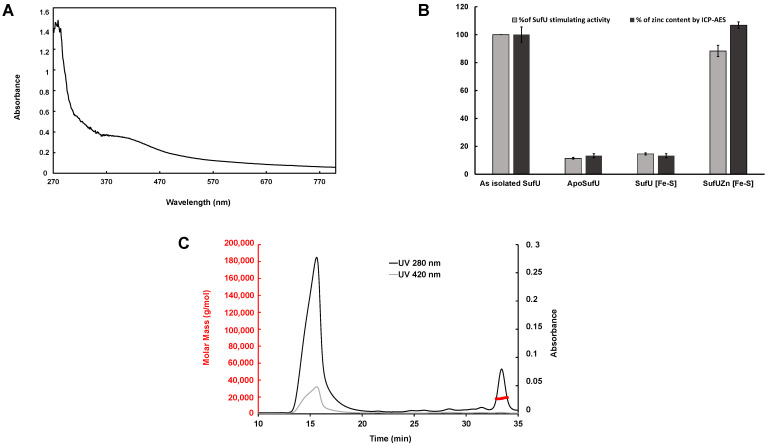
(**A**) UV-Vis absorption spectrum of apo-SufU (1 mg/mL) reconstituted with Fe^2+^, catalytic SufS, and L-cysteine, showing spectral features of an [Fe-S] cluster-containing protein. (**B**) Sulfurtransferase activity of SufU (gray bars) under different forms (as-isolated, apo-form, Zn-reconstituted form, [Fe-S] reconstituted from the apo-SufU, and Zn-SufU reconstituted with [Fe-S]). Zinc content (%) was measured for the different SufU forms by ICP-AES (black bars). (**C**) SEC-MALLS of apo-SufU reconstituted with Fe^2+^ in the presence of catalytic SufS and L-cysteine.

As mentioned previously, SufU from *B. subtilis* is reported to bind a [2Fe-2S] cluster substoichiometrically after protein purification and in higher amounts after reconstitution [23]. This form can transfer its cluster to the Leu1 protein. In contrast, another study claimed that *B. subtilis* could not act as a standard Fe-S scaffold protein [22,60]. *E. faecalis* SufU was also reported to bind an Fe-S cluster [61]. Based on these contrasting results, we decided to investigate *Mtb* SufU as a potential Fe-S binding protein. We prepared *Mtb* apo-SufU (0.1 Zn/protein) and incubated it anaerobically with an excess of ferrous iron, L-cysteine, and a catalytic amount of *Mtb* SufS. After desalting, the protein displayed a UV-vis. spectrum characteristic of a [4Fe-4S] cluster and contained 4.01 Fe and 3.37 S/monomer (Figure 12A). The reconstituted Fe-S SufU protein had the same SufS activation capacity as the apo-SufU (10%). This residual activity is likely due to the remaining zinc bound to the protein (Figure 12B). The chromatogram of the reconstituted SufU protein after SEC-MALLS performed under anaerobic conditions revealed the presence of two peaks (Figure 12C). Peak 1 (major) corresponds to oligomeric states of SufU, whereas peak 2 (minor) corresponds to the monomeric form of SufU. Absorbance at 420 nm, characteristic of the presence of an Fe-S cluster, was also measured and revealed Fe-S cluster(s) to only be associated with oligomeric states of SufU. This result suggests that the Fe-S clusters present are non-specific.

Since purified Mtb SufU binds a zinc ion, we next wondered whether Zn-SufU could be an intermediate in the formation of an Fe-S on SufU. We incubated as-isolated Zn-SufU (1 zinc/monomer) with catalytic amounts of 6xHisSufS, L-cysteine, and ferrous iron. After passage on a Ni-NTA column to remove SufS, SufU contained 3.4 Fe, 2.7 S, and an unchanged 1.1 Zn/ monomer (Table 3), indicating that zinc was not replaced by the Fe and S treatment. These data indicate that the zinc-binding site does not contain an Fe-S cluster, suggesting once again that any binding of Fe and S is aspecific. In line with this conclusion, the sulfurtransferase activity measured for this protein was close to the as-isolated SufU (95%). Altogether, these data show that the sulfurtransferase activity of SufU requires zinc rather than an Fe-S cofactor, strongly suggesting that Mtb SufU is not an Fe-S protein.

### 3.9. Mtb SufS-SufU Is More Resistant to H_2_O_2_ Than E. coli SufS-SufE

Both in vivo and in vitro data on *E. coli* SUF support the idea that the SUF system is functional under oxidative stress conditions [56,63]. This makes sense for *Mtb*, which is permanently exposed to this type of stress during infection when it encounters reactive oxygen and nitrogen species produced by macrophages [63,64]. Intriguingly, some differences exist between the *Mtb* and *E. coli* SUF systems, in particular with respect to the sulfur mobilization system. In *E. coli*, this system is composed of SufS and SufE, whereas in *Mtb,* it consists of SufS and SufU. Moreover, SufU is a metalloprotein, whereas SufE is not. Does the presence of zinc in SufU make the *Mtb* SufS-SufU system more resistant to oxidative stress than the *E. coli* SufS-SufE system?

**Figure 13:**
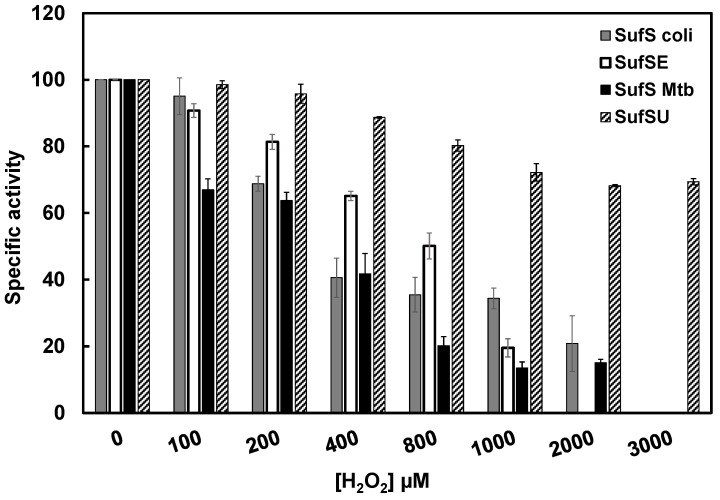
Sensitivity of *E. coli* SufS-SufE and *Mtb* SufS-SufU to H_2_O_2_ during the cysteine-desulfuration reaction. SufS-SufE (1:20) and SufS-SufU (1:80) proteins (*Mtb* and *E. coli* SufS: 0.5 µM) were mixed for 5 min. Then, 500 µM L-cysteine was added to initiate the reaction, followed immediately by the addition of 0–3 mM H_2_O_2_. Samples were incubated for 30 min before quenching the reaction by heating to 95 °C for 5 min. The addition of 1 mM DTT reduced the samples, releasing sulfide for measurement. All steps were carried out in anaerobic conditions. Percent activity of SufS (gray bar), SufS-SufE (white bar), *Mtb* SufS (black bar), and SufS-SufU (hatched bar) compared to their activity without H_2_O_2_ (100%).

To test this hypothesis, we compared sulfur production by *Mtb* SufS with its sulfurtransferase SufU to that of *E. coli* SufS with its sulfurtransferase SufE in oxidative stress conditions by assessing their relative in vitro H_2_O_2_ sensitivity. Since it is difficult to test for H_2_O_2_ sensitivity in the presence of DTT due to the propensity for DTT to react with and consume H_2_O_2_ and its ability to reverse some H_2_O_2_-mediated thiol oxidation products, such as sulfenic acid [65], these experiments were performed without DTT, under anaerobic conditions (Figure 13). We observed a pronounced difference in H_2_O_2_ sensitivity between the *E. coli* SufSE and *Mtb* SufSU systems (Figure 13). *E. coli* SufSE activity was reduced by 35% at 400 µM H_2_O_2_, as reported elsewhere [56]. In contrast, *Mtb* SufSU activity was only 10% lower at this H_2_O_2_ concentration (Figure 13). At 1 mM H_2_O_2_, SufSE was 80% inactivated, whereas SufSU activity was reduced by just 30%. Interestingly, both *Mtb* SufS and *E. coli* SufS showed similarly reduced activity levels following H_2_O_2_ treatment, indicating that it was the presence of SufU that significantly changed the H_2_O_2_ sensitivity of the *Mtb* SufS activity. This result suggests that the higher H_2_O_2_ tolerance of the *Mtb* SufS-SufU system is due to the zinc in SufU.

The higher resistance of *Mtb* SufSU to H_2_O_2_ might be the result of less oxidation of cysteine residues. Oxidation of cysteine thiols by H_2_O_2_ yields sulfenic acid (S-OH), which can react with a neighboring cysteine thiol to generate a disulfide bond [66,67]. Further oxidation can also lead to sulfinic acid formation [68]. We investigated disulfide bond formation in *Mtb* SufS and SufU following H_2_O_2_-mediated oxidation of their cysteine thiolates. We also tested *E. coli* SufS and SufE. The formation of disulfides in each oxidized sample was qualitatively analyzed by SDS-PAGE. After treatment with 1 mM H_2_O_2_, samples were separated by SDS-PAGE under both reducing (+DTT) and non-reducing (−DTT) conditions (Figure S8). Regardless of H_2_O_2_ treatment, *E. coli* SufS alone always migrated at its expected monomeric molecular weight, suggesting an absence of disulfide bond formation in these conditions. Similarly, *E. coli* SufE formed no disulfide-bonded homodimers in the absence of H_2_O_2_ (Figure S8). The relative amount of SufE homodimer increased from 0% to 34% of the total SufE protein upon exposure to H_2_O_2_, as previously observed [55]. In samples containing both SufE and SufS, SufE homodimers were observed in similar amounts (Figure S8), indicating that *E. coli* SufS does not protect SufE cysteines from oxidation. No higher molecular species corresponding to covalent SufS-SufE heterodimers were observed.

Similar to *E. coli* SufS, *Mtb* SufS did not form a disulfide bond in the presence of H_2_O_2_ (Figure S8), suggesting that H_2_O_2_ inhibits both SufS enzymes through another mechanism. In contrast to SufE, *Mtb* SufU did not form homodimers in the presence of H_2_O_2_ (Figure S8). When *Mtb* SufS was mixed with SufU, a band at around 72 kDa that may correspond to a SufS-SufU heterodimer was observed (Figure S8). Importantly, no disulfide-bonded SufU homodimers were observed. Taken together, these results show that upon exposure to H_2_O_2_, *Mtb* SufU has no propensity to form covalently-linked dimers, unlike *E. coli* SufE. This behavior provides a mechanism ensuring that *Mtb* SufS-SufU is less inhibited by exposure to H_2_O_2_ than *E. coli* SufS-SufE.

## 4. Discussion

In this paper, we present the first characterization of two proteins from the Mtb suf operon: RV1464 (SufS) and Rv1465 (SufU). In our study, we used biochemical and structural approaches to obtain a good understanding of how these proteins work. The results presented can be used in the future to develop molecules to inactivate these proteins as a means to combat tuberculosis infections.

Our results revealed SufS to be a type II cysteine desulfurase. Indeed, Mtb SufS displays a low basal cysteine-desulfurase activity (5 nmol/min/mg). The slow cleavage of persulfide allowed us to calculate the number of active sites. Our results suggest that only half of the enzyme is active. A similar feature was observed for *E. coli* and *B. subtilis* SufS [13,21]. It is possible that activation of one site provokes structural changes in the other subunit, creating a negative cooperative regulation between the two subunits in each dimer. Recent structural studies on SufS from *E. coli* support this hypothesis, with conformational changes in one subunit upon persufuration of the other (Frantom et al. 2022, personal communication). This type of behavior of SufS might be a way for the enzyme to control the sulfur transfer reaction.

Another trademark of class II cysteine desulfurases is their activation by sulfurtransferase. In our case, Mtb SufS activity was significantly enhanced in the presence of stoichiometric amounts of the sulfurtransferase SufU (59 nmol/min/mg). Moreover, enzymatic studies revealed Mtb SufU to be a substrate of Mtb SufS, in addition to L-cysteine. Under optimal conditions, with 80 equivalents of SufU and 100 equivalents of L-cysteine, a velocity of about 900 nmol/min/mg was obtained, which is among the highest for cysteine desulfurases (Table 4). So far, three SUF systems containing a SufU protein have been characterized, namely *B. subtilis*, *S. aureus*, and *E. faecalis* (Table 4). The Mtb SufS-SufU system is very similar to the *B. subtilis* system, with SufU as a substrate of SufS and inducing a >100-fold stimulating effect [21]. In the *E. faecalis* system, SufU also stimulates SufS activity, but to a lesser extent (Table 4) [62]. Surprisingly, SufS from *S. aureus* was recently reported to be poorly stimulated in the presence of its cognate SufU [61]. It is possible that the *S. aureus* system requires SufBCD in addition to SufU to stimulate SufS, as observed for the *E. coli* system [16].

Our results show that *Mtb* SufU sulfurtransferase activity is zinc-dependent. *Mtb* SufU was purified with one zinc ion/ monomer that binds the protein with high affinity (Ka = 4.23 × 10^−^^16^ M^−^^1^). Removal of the zinc produced an apo-protein that failed to display a sulfurtransferase activity. Moreover, our results show that this activity (as measured by monitoring sulfur production) correlated directly with the amount of zinc associated with SufU. Apo-SufU could be reactivated after reconstitution with Zn^2+^ or simply by incubation with zinc salts.

In the literature, some proteins involved in Fe-S biogenesis or in other processes can be purified with Zn, even though the physiological metal is Fe [69,70]. Our results demonstrate that other divalent metal ions cannot replace zinc and produce equivalent activity, although Co^2+^ allowed SufU to promote 50% SufS activation. Partial activity with Co^2+^ is often observed with known zinc proteins [57,58,59] and can be explained by the similar electronic configuration of these two transition metals. Despite the partial activity with Co^2+^, the low sulfurtransferase activity observed in the presence of other metal ions strongly suggests that Mtb SufU sulfurtransferase activity is zinc-specific. It is worth noting that two other SufU proteins have also been purified with zinc, SufU from *B. subtilis* and from *S. aureus* [22,61].

The crystal structure of the Mtb SufS_2_-SufU_2_ complex revealed that the zinc ion is tetracoordinated by one ligand of SufS (His354) and three ligands of SufU (Cys31, Asp42, and Cys67). Zinc was observed to play a crucial role in mediating the SufS-SufU interaction. His354 is essential for the sulfurtransferase activity, as shown in the cross-species experiment associating Mtb SufU with *E. coli* SufS, which contains a tyrosine in its sequence instead of a histidine residue. In this system, no sulfurtransferase activity was detected. The zinc coordination mode, involving the SufS protein, ensures that the conserved SufU Cys40 faces the SufS Cys373, making it accessible for the sulfurtransferase reaction between SufS and SufU. This result is in agreement with observations from *B. subtilis*, where the equivalent conserved Cys41 corresponds to the persulfuration site, but in contrast to results from *E. faecalis*, where Cys128 on SufU was proposed as the sulfur acceptor site [62]. Mechanistically, since SufU is a substrate of SufS, this implies that once persulfurated (SufU_Cys40-SSH_), SufU dissociates from SufS, allowing another SufU to interact with it and to be persulfurated. In this context, when the persulfurated SufU dissociates from SufS, two hypotheses can be proposed: either Cys40-SSH could coordinate once again with the zinc atom, or a water molecule could take the place of the fourth ligand, leaving Cys40-SSH exposed to the solvent. No evidence of a persulfurated cysteine coordinating zinc has ever been presented. In the presence of the scaffold, it is likely that sulfur from Cys40-SSH is directly transferred, allowing Cys40 to coordinate the zinc ion once again.

What role does the zinc ion play in Mtb SufU, since there is no metal in *E. coli* SufE sulfurtransferase? The role of the zinc ion might be to protect Cys40 from oxidation in the absence of SufS. Results from experiments where proteins were exposed to H_2_O_2_, monitored by SDS-gel under non-reducing conditions, support this hypothesis. Disulfide-bonded SufU homodimers were not formed, but SufE homodimers were. When SufU interacts with SufS, only SufS-SufU heterodimers appear to form, in agreement with Cys40 becoming accessible and reacting with the nearby SufS Cys373. In contrast, SufE homodimers are still formed in the presence of SufS, suggesting that the presence of SufS does not protect SufE cysteines (in particular, the catalytic Cys41) from oxidation. This difference in cysteine-oxidation patterns between the two systems might explain the better resistance to oxidative stress of the Mtb SufSU system compared to the *E. coli* SufSE system. This enhanced resistance allows continued sulfur production despite the continual oxidative stress to which Mtb is exposed due to its route of infection. One can hypothesize that the high tolerance to H_2_O_2_ is a strategy adopted by Mtb to better resist infection.

The literature contains contradictory results regarding the presence of an Fe-S cluster within SufU proteins. The *E. faecalis* SufUD37A variant was isolated with an Fe-S cluster [62], and *B. subtilis* SufU was reported to bind an Fe-S cluster after reconstitution [23]. However, another study on *B. subtilis* claims that the association between Fe-S and SufU is artifactual [22]. Recently, SufU from *S. aureus* was characterized as a zinc-binding protein unable to bind an Fe-S cluster [61]. Using both Mtb Zn-SufU and apo-SufU proteins, we detected no Fe-S clusters bound to the active site, strongly suggesting that SufU is not an Fe-S protein/scaffold. This makes sense, as the Mtb suf operon includes SufB, SufC, and SufD, which likely play this role by analogy with the *E. coli* system [18,19].

In conclusion, the results from this work provide the basis for the investigation of SufS and SufU as potential targets for antibacterial agents targeting Mtb.

## Figures and Tables

**Table 1:** Concentration of metals in as-purified *Mtb* SufU determined by ICP-AES. Experiments were performed in triplicate.

Element	Concentration (µM/µM as-Isolated SufU)
Fe	0
Ni	0.1
Zn	1.1 ± 0.1

Fe: iron; Ni: nickel; Zn: zinc.

**Table 2:** Kinetic constants of the SufS reaction.

Substrates	K_m_ (µM)	V_max_ (nmol/min/mg)	kcat (s^−1^)	kcat/K_m_ (s^−1^ M^−1^)
Cysteine	103	1250	0.94	9.1 × 10^3^
SufU	7.8	909	0.68	8.7 × 10^4^

**Table 3:** Metal content of *Mtb* Zn-SufU after reconstitution with Fe, SufS, and L-cysteine.

Element	Concentration (µM/µM Protein)
Fe	3.4 ± 0.14
S	2.7 ± 0.18
Zn	1.1 ± 0.10

Zinc content was determined by ICP-AES, and sulfur content by methylene blue formation. Iron was quantified by both ICP-AES and biochemical methods.

**Table 4:** Comparison of the activities of several cysteine desulfurases. Type I: IscS; type II: SufS from *E. coli*, *B. subtilis*, *S. aureus*, *E. faecalis*, and *Mtb*. Stimulatory effect of type II cysteine desulfurases in the presence of their respective sulfurtransferase. (/): not concerned.

Cysteine Desulfurase	IscS *E. coli*	SufSE *E. coli*	SufSU *B. subtilis*	SufSU *S. aureus*	SufSU *E. faecalis*	SufSU Mtb
Velocity (nmol/min/mg)	90	600	1000	6	150	900
Stimulating factor	/	150	40–130	1.5	37	180

## Data Availability

Structure of SufU from *Bacillus subtilis* (PDB number: 6JZV).

## Supplementary Materials

The following supporting information can be downloaded at: https://www.mdpi.com/article/10.3390/biom13050732/s1, Figure S1: Specific activity of *Mtb* SufS in the presence of *E. coli* SufE or *Mtb* SufU; Figure S2: SufS-SufU interaction in solution; Figure S3: Reconstitution of apo-SufU with zinc; Figure S4: Sulfurtransferase activity of SufU; Figure S5: Crystal structure of the *Mtb* SufS-SufU complex; Figure S6: Sequence alignment of type I and type II cysteine desulfurases; Figure S7: Specific activity of *E. coli* SufS with *E. coli* SufE and *Mtb* SufU; Figure S8: SDS-PAGE gel of H_2_O_2_-treated *E. coli* and *Mtb* SufS and SufU proteins; Table S1: X-ray data and refinement statistics; Table S2: Contact interactions of *Mtb* SufS and SufU [71].
