# Supplementary material for: Structural and Biochemical Characterization of Mycobacterium tuberculosis Zinc SufU-SufS Complex
Source: Biomolecules. 2023 Apr 24;13(5):732. doi: 10.3390/biom13050732 (PMC10216065; doi:10.3390/biom13050732)
Supplement: Supplementary file 1 [file biomolecules-13-00732-s001.zip › biomolecules-2282495-supplementary.pdf]

SUPPLEMENTARY MATERIALS

Supplementary Methods

SufS and SufU interaction

6xHis-SufU was incubated for 10 min. with SufS (in stochiometric amount) and nickel nitrilotriacetic acid (Ni-NTA) resin with and without 5 mM L-cysteine. The mix was then loaded on a column and was left to flow by gravity without any pressure applied. The column was then washed with one column volume of buffer J. Bound proteins containing 6xHis-SufU and the 6xHis-SufU/SufS complex were eluted with buffer J containing 250 mM imidazole. The flow-through and the eluted proteins were analyzed by SDS-PAGE. Based on the SDS-PAGE profile, the concentration of 6xHis-SufU in the flow-though was assumed to be neglected so the protein content in the flow-through was assumed to be the amount of SufS that does not carry a 6xHis-tag and that didn't interact with 6xHis-SufU. The amount of SufS that interacted with SufU was quantified by simple deduction of the amount of SufS quantified in the flow-through from the initial amount of SufS that was used in the experiment.

Supplementary Results

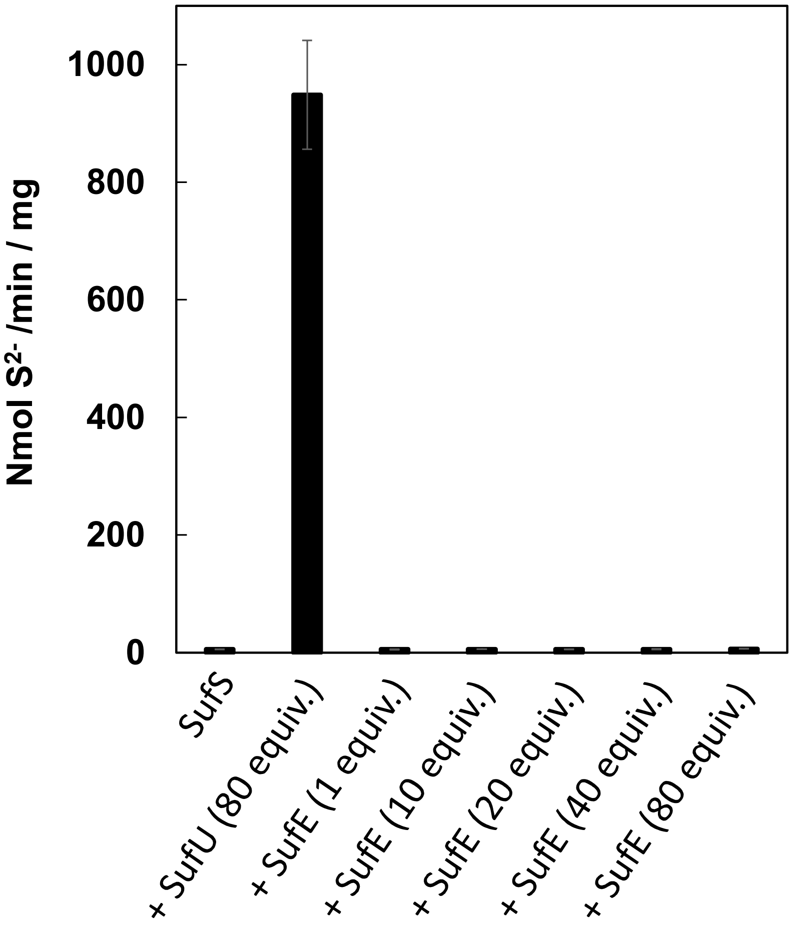

Figure S1. Specific activity of *Mtb* SufS (10 μM) in the absence and in the presence of different equivalents of *E. coli* SufE (1-80 equivalents) or *Mtb* SufU (80 equivalents). Cysteine at 500 μM was used in the assay.

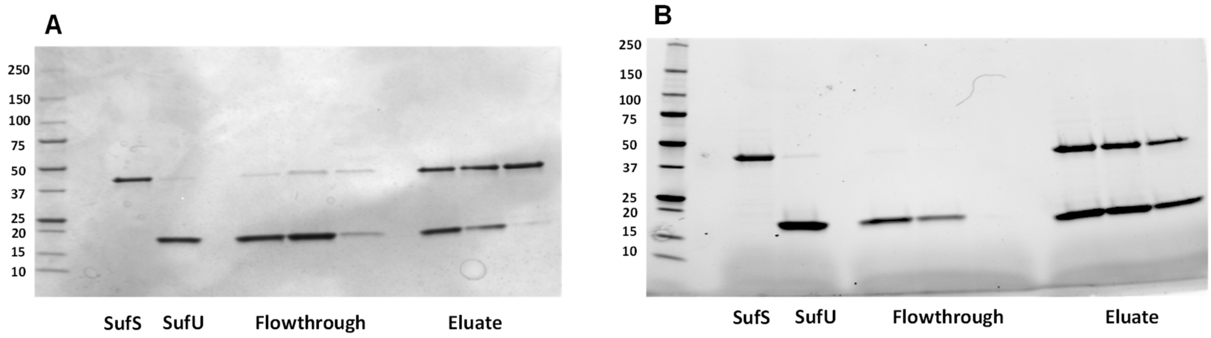

Figure S2. (A) SufS-SufU interaction in solution. (A) SDS-PAGE profile of 6xHis-SufS and SufU interaction in the absence of L-cysteine on affinity column (Ni-NTA). Lane (1) : known size markers (kDa), lane (2) : purified 6xHis-SufS, lane (3): purified SufU, lanes (4-6) flowthrough (FT) samples containg fractions of SufU which doesn't interact with SufS, lanes (7-9): eluate samples containg 6xHis-SufS and SufU. Protein quantification: FT: 63.8% of total SufU; Eluate: 36.2% of SufU. (**B)** SDS-PAGE profile of 6xHis-SufS and SufU interaction in the presence of 5mM L-cysteine on affinity column (Ni-NTA). Lane (1) : known size markers (kDa), lane (2) : purified 6xHis-SufS, lane (3): purified SufU, lanes (4-6) flowthrough (FT) samples containg fractions of SufU which doesn't interact with SufS, lanes (7-9): eluate samples containg 6xHis-SufS and SufU. Protein quantification: FT: 22% of total SufU; Eluate: 78% of SufU. From the quantitation, it seems that the interaction is increased in the presence of L-cysteine.

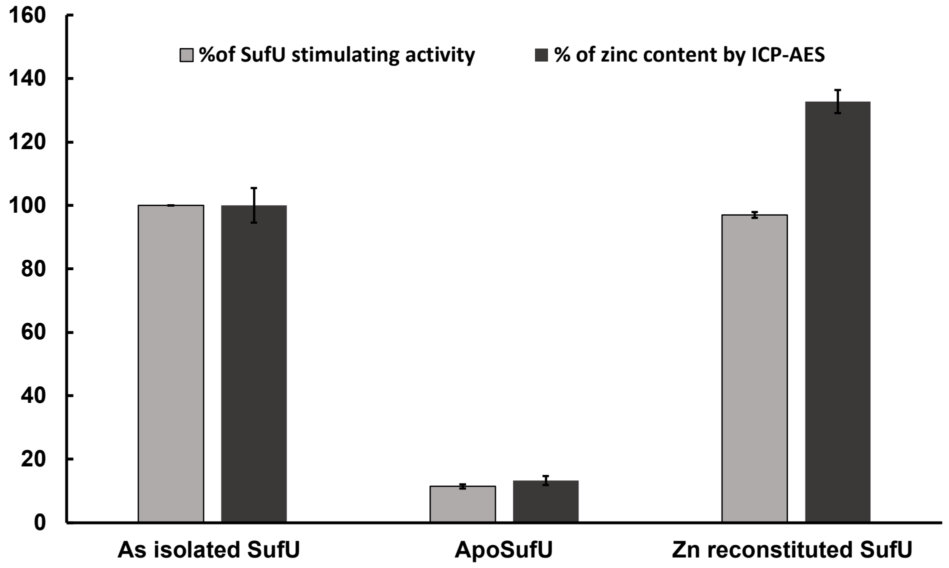

Figure S3. Reconstitution of apo-SufU with zinc. The SufU stimulating activity (%) was determined by measuring the specific activity of SufS (10 μM) in presence of as-isolated SufU (10 μM), apo-SufU (10 μM) and Zn reconstituted SufU (10 μM). The sulfurtransferase activity with the as-isolated SufU (60 nmoles S^{2-}/min/mg) was considered as 100%. Apo-SufU displays an activity of 11.4% (6.4 nmoles S^{2-}/min/mg), while SufU reconstituted with zinc was able to restore 96% (57.7 nmoles S^{2-}/min/mg) of the as-isolated SufU sulfurtransferase activity. The zinc content was quantified by ICP-AES. The 100% content corresponds to 1.1 Zn/ monomer of the as-isolated SufU. The zinc content in apo-SufU is 13.2% (0.145 Zn/ monomer) and in case of the Zn reconstituted SufU, the zinc content is 132% (1.46 Zn/monomer) reflecting some unspecific zinc.

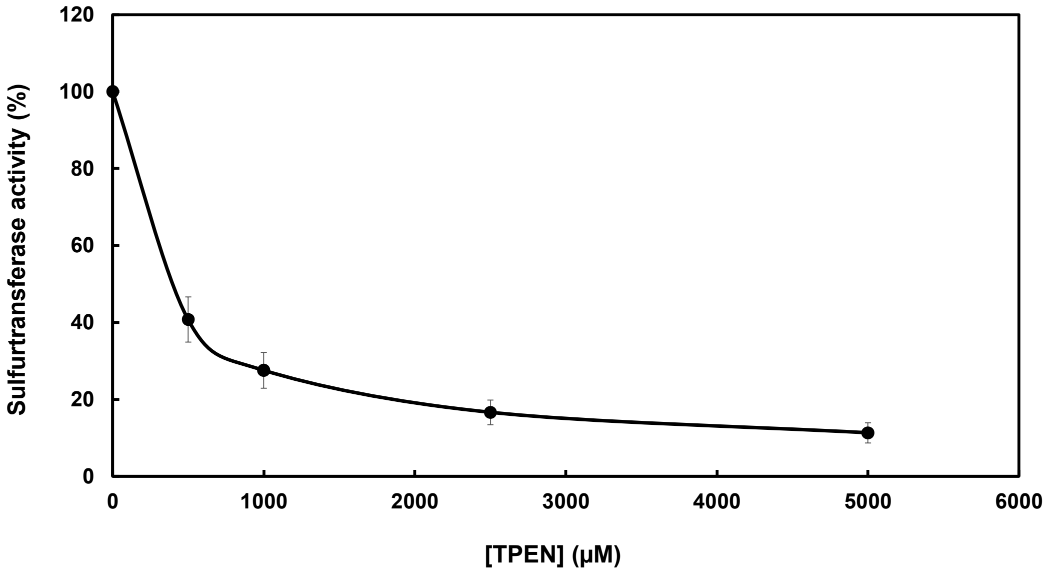

Figure S4. Sulfurtransferase activity of SufU (%). SufU (146 µM) was incubated with increasing concentrations of TPEN (0-5 mM) for 3 h followed by dialysis. The sulfur transfer activity of SufU was then determined by measuring the specific activity of SufS (10 µM) in the presence of each sample (10 µM), L-cysteine (500 µM), DTT (2 mM) for 30 min at 37°C. 100% corresponds to 78 nmoles of S^{2-}/min/mg of SufS. The curve is superposable to that in Figure 5 showing that the loss of SufU sulfurtransferase activity is due to the gradual decrease in zinc content by increasing TPEN concentration.

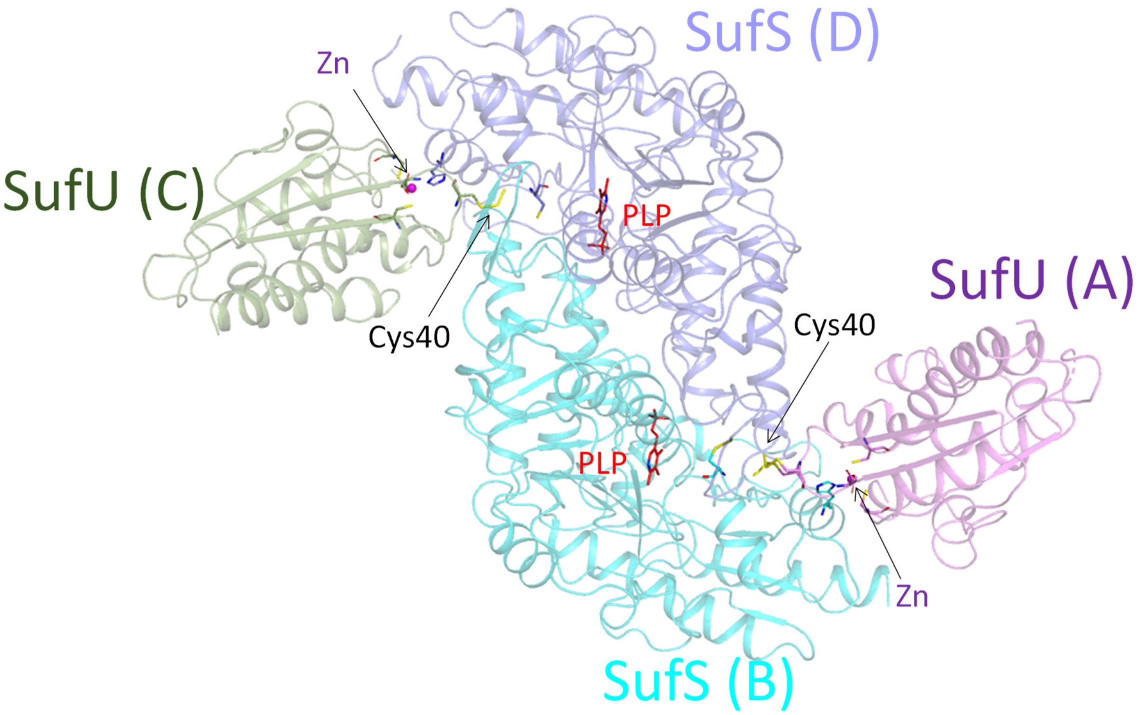

Figure S5. Overall crystal structure of *Mtb* SufS-SufU complex. The complex is represented in cartoon representation. The figure shows the arrangement of the different subunits, which consists as a dimer of complexes SufS-SufU (SufU in green and violet, SufS in cyan and blue). The PLP cofactor is represented in red inside SufS, and the zinc ions in purple at the interface SufS-SufU with their environment drawn in stick representation.

```
CsdA_Ecoli          -----MNVFNPAQFRAQFPALQD------AGVYLDSAATALKPEAVVEATQQFYSLS--A     47
Rv1464_Mtub         -MTASVNSLDLAAIRADFPILKRIMRGGNPLAYLDSGATSQRPLQVLDAEREFLTAS--N     57
SufS_Saureus        MNEVAEHSFDVNEVIKDFPILDQKVN-GKRLAYLDSTATSQTPVQVLNVLEDYYKRY--N     57
SufS_Bsubtilis      --------MNITDIREQFPILHQQVN-GHDLVYLDSAATSQKPRAVIETLDKYYNQY--N     49
SufS_Efaecalis*     -------MMDAATIRQSFPILFQEVN-DEPLVYLDNAATTQKTTAVLDVLRHYYETD--N     50
SufS_Ecoli          ------MIFSVDKVRADFPVLSREVN-GLPLAYLDSAASAQKPSQVIDAEAEFYRHG--Y     51
SufS_MXAN           -----MSGFDVNQVRKDFPILDQEVR-GRPLVYLDSAATAQKPQAVIDALVRFYQHD--N     52
                                                    ***  *::     * :    :

IscS_Ecoli          GNPASRSHRFGWQAEEAVDIARNQIADLVGA-DPREIVFTSGATESDNLAIKGAANF---     89
CsdA_Ecoli          GNVHRSQFAEAQRLTARYEAAREKVAQLLNAPDDKTIVWTRGTTESINMVAQCYARP---     104
Rv1464_Mtub         GAVHRGAHQLMEEATDAYEQGRADIALFVGA-DTDELVFTKNATEALNLVSYVLGDSRFE     116
SufS_Saureus        SNVHRGVHTLGSLATDGYENARETVRRFINAKYFEEIIFTRGTTASINLVAHSYGDA---     114
SufS_Bsubtilis      SNVHRGVHTLGTRATDGYEGAREKVRKFINAKSMAEIIFTKGTTTSLNMVALSYARA---     106
SufS_Efaecalis*     ANVHRGVHTLAERATKDYEASREKVRQFIHAKETAEVLFTRGTTTSLNWIAKSYGDL---     107
SufS_Ecoli          AAVHRGIHTLSAQATEKMENVRKRASLFINARSAEELVFVRGTTEGINLVANSWGNS---     108
SufS_MXAN           ANVHRGVHVLSERATEAYEGARETVRRFINARDVKEVVFVRGTTEAINLVAQTYGRK---     109
                    .      .          :  *     :: *     :::. .:* . *      .

IscS_Ecoli          -YQKKGKHIITSKTEHKAVLDTCRQL-EREGFEVTYLAPQRNGIIDLKELEAAMRDDTIL     147
CsdA_Ecoli          -RLQPGDEIIVSVAEHHANLVPWLMVAQQTGAKVVKLPLNAQRLPDVDLLPELITPRSRI     163
Rv1464_Mtub         RAVGPGDVIVTTELEHHANLIPWQELARRTGATLRWYGVTDDGRIDLDSL--YLDDRVKV     174
SufS_Saureus        -NVEEGDEIVVTEMEHHANIVPWQQLAKRKNATLKFIPMTADGELNIEDIKQTINDKTKI     173
SufS_Bsubtilis      -NLKPGDEVVITYMEHHANIIPWQQAVKATGATLKYIPLQEDGTISLEDVRETVTSNTKI     165
SufS_Efaecalis*     -AVTAGDEIVISYMEHHSNIIPWQQLAQRTGAILKYIDVTEDGFLDMASARQQITEKTKI     166
SufS_Ecoli          -NVRAGDNIIISQMEHHANIVPWQMLCARVGAELRVIPLNPDGTLQLETLPTLFDEKTRL     167
SufS_MXAN           -HIGAGDEVLITQMEHHANIVPWRMLCEQTGAVLKVIPVDDRGELVLDAVDALLTERTRI     168
                         *. :: :  **:: :          .  :            :      .     :

IscS_Ecoli          VSIMHVNNEIGVVQDIAAIGEMCRARGIIYHVDATQSVGKLPIDLSQLKVDLMSFSGHKI     207
CsdA_Ecoli          LALGQMSNVTGGCPDLARAITFAHSAGMVVMVDGAQGAVHFPADVQQLDIDFYAFSGHKL     223
Rv1464_Mtub         VAFTHHSNVTGVLTPVSELVSRAHQSGALTVLDACQSVPHQPVDLHELGVDFAAFSGHKM     234
SufS_Saureus        VAIAHISNVLGTINDVKTIAEIAHQHGAIISVDGAQAAPHMKLDMQEMNADFYSFSGHKM     233
SufS_Bsubtilis      VAVSHVSNVLGTVNPIKEMAKIAHDNGAVIVVDGAQSTPHMKIDVQDLDCDFFALSSHKM     225
SufS_Efaecalis*     VSIAHVSNVLGVINPIEELTQLAHQNGAVMVVDGAQAVPHMPVDVQAIDADFYAFSGHKM     226
SufS_Ecoli          LAITHVSNVLGTENPLAEMITLAHQHGAKVLVDGAQAVMHHPVDVQALDCDFYVFSGHKL     227
SufS_MXAN           LAVTHVSNALGTVAPVKELTRRAHAKGIPVLVDGAQAVTHFPVDVQDLGCDFYAFSGHKM     228
                    ::. : .*   *    :      .:  *    :*. *.. :   *:  :  *:  :*.**:

IscS_Ecoli          YGPKGIGALYVRRKPRVRIEAQMHGGGHER---------------GMRSGTLPVHQIVGM     252
CsdA_Ecoli          YGPTGIGVLYGKSELLEAMSPWLGGGKMVHEVSF-DGFTTQSAPWKLEAGTPNVAGVIGL     282
Rv1464_Mtub         LGPNGIGVLYGRRELLAQMPPFLTGGSMIETVTM-EGATYAPAPQRFEAGTPMTSQVVGL     293
SufS_Saureus        LGPTGIGVLFGKRELLQKMEPIEFGGDMIDFVSK-YDATWADLPTKFEAGTPLIAQAIGL     292
SufS_Bsubtilis      CGPTGVGVLYGKKALLENMEPAEFGGEMIDFVGL-YESTWKELPWKFEAGTPIIAGAIGL     284
SufS_Efaecalis*     CGPTGIGVLYGKRHLLEQMEPVEFGGEMIDFVHL-QESTWKELPWKFEAGTPNIAGAIAL     285
SufS_Ecoli          YGPTGIGILYVKEALLQEMPPWEGGGSMIATVSLSEGTTWTKAPWRFEAGTPNTGGIIGL     287
SufS_MXAN           FGPTGIGVLYGRKERLDAMPPYQGGGDMILSVTM-EKVTYNRVPYRFEAGTPNLEGAVGL     287
                     **.*:* *: :      :     **                    :.:**      :.:

IscS_Ecoli          GEAYRIAKEEMATEME-RLRGLRNRLWNGIKDIEEVYLNGDL---EHGAPNILNVSFNYV     308
CsdA_Ecoli          SAALEWLADYDINQAESWSRSLATLAEDALAKRPGFRSFRCQ----DSSLLAFDFAGVHH     338
Rv1464_Mtub         AAAARYLGAIGMAAVEAHERELVAAAIEGLSGIDGVRILGPTSMRDRGSPVAFVVEGVHA     353
SufS_Saureus        AEAIRYLERIGFDAIHKYEQELTIYAYEQMSAIEGIEIYGPPKD-RRAGVITFNLQDVHP     351
SufS_Bsubtilis      GAAIDFLEEIGLDEISRHEHKLAAYALERFRQLDGVTVYGPE---ERAGLVTFNLDDVHP     341
SufS_Efaecalis*     GAAIDYLTEIGLEAIHQHEAALVHYVLPKLQAIEGLTIYGPQNPKDHTGVIAFNIEGLHP     345
SufS_Ecoli          GAALEYVSALGLNNIAEYEQNLMHYALSQLESVPDLTLYGPQ---NRLGVIAFNLGKHHA     344
SufS_MXAN           AAAIRYLEALGMENVAAHDRELLAYATQALESVPGLRMVGTAR--EKSGVLSFMLADIHP     345
                    . *                  *       :     .            .   : .   :

IscS_Ecoli          EGESLIMALKDLAVSSGSACTSASLEPSYVLRALGLNDELAHSSIRFSLGRFTTEEEIDY     368
CsdA_Ecoli          SDMVTLLAEYGIALRAGQHCAQPLLA------ELGV-----TGTLRASFAPYNTKSDVDA     387
Rv1464_Mtub         HDVGQVLDDGGVAVRVGHHCALPLHR------RFGL-----AATARASFAVYNTADEVDR     402
SufS_Saureus        HDVATAVDTEGVAVRAGHHCAQPLMK------WLNV-----SSTARASFYIYNTKEDVDQ     400
SufS_Bsubtilis      HDVATVLDAEGIAVRAGHHCAQPLMK------WLDV-----TATARASFYLYNTEEEIDK     390
SufS_Efaecalis*     HDVATALDMEGVAVRAGHHCAQPLLN------YLSV-----PATARASFYLYNTKEDADR     394
SufS_Ecoli          YDVGSFLDNYGIAVRTGHHCAMPLMA------YYNV-----PAMCRASLAMYNTHEEVDR     393
SufS_MXAN           HDVGTILDREGICIRTGHHCAQPVMQ------HFKV-----PATSRASLALYNTREDVDA     394
                     .    :   .:.:  *  *:              :      .  * *:  :.* .: *

IscS_Ecoli          TIELVRKSIGRLRDLSPLWEMYKQGVDLNSIEWAHH 404
CsdA_Ecoli          LVNAVDRALELLVD---------------------- 401
Rv1464_Mtub         LVAGVRRSRHFFGRA--------------------- 417
SufS_Saureus        LINALKQTKEFFSYEF-------------------- 416
SufS_Bsubtilis      LVEALQKTKEYFTNVF-------------------- 406
SufS_Efaecalis*     LVEAIKATKEFFQHGTF------------------- 411
SufS_Ecoli          LVTGLQRIHRLLG----------------------- 406
SufS_MXAN           LVRGLHKVLEVFQ----------------------- 407
```

Figure S6. Sequence alignment of type I and type II cysteine desulfurases. Type I (*E. coli* IscS) and type II (*E. coli* CsdA, *E. coli* SufS, *S. aureus* SufS, *B. subtilis* SufS, *E. faecalis* SufS, *Myxococcus xanthus* (MXAN) SufS and *Mtb* SufS). Highlight yellow : conserved histidine residue in type II cysteine desulfurases SufS functioning with SufU as sulfurtransferase.

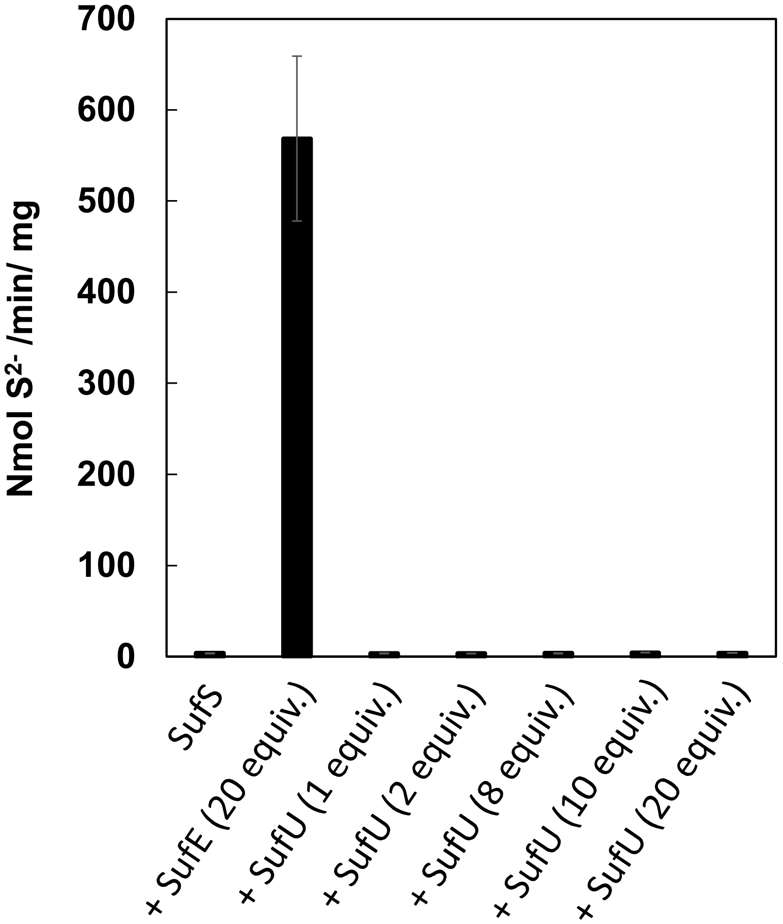

Figure S7. Specific activity of *E. coli* SufS (10 µM) in the absence and in the presence of different equivalents of *E. coli* SufE (20 equivalents) and *Mtb* SufU (1-20 equivalents) for 30 min. at 37°C in presence of L-cystéine (500 µM) and DTT (1 mM).

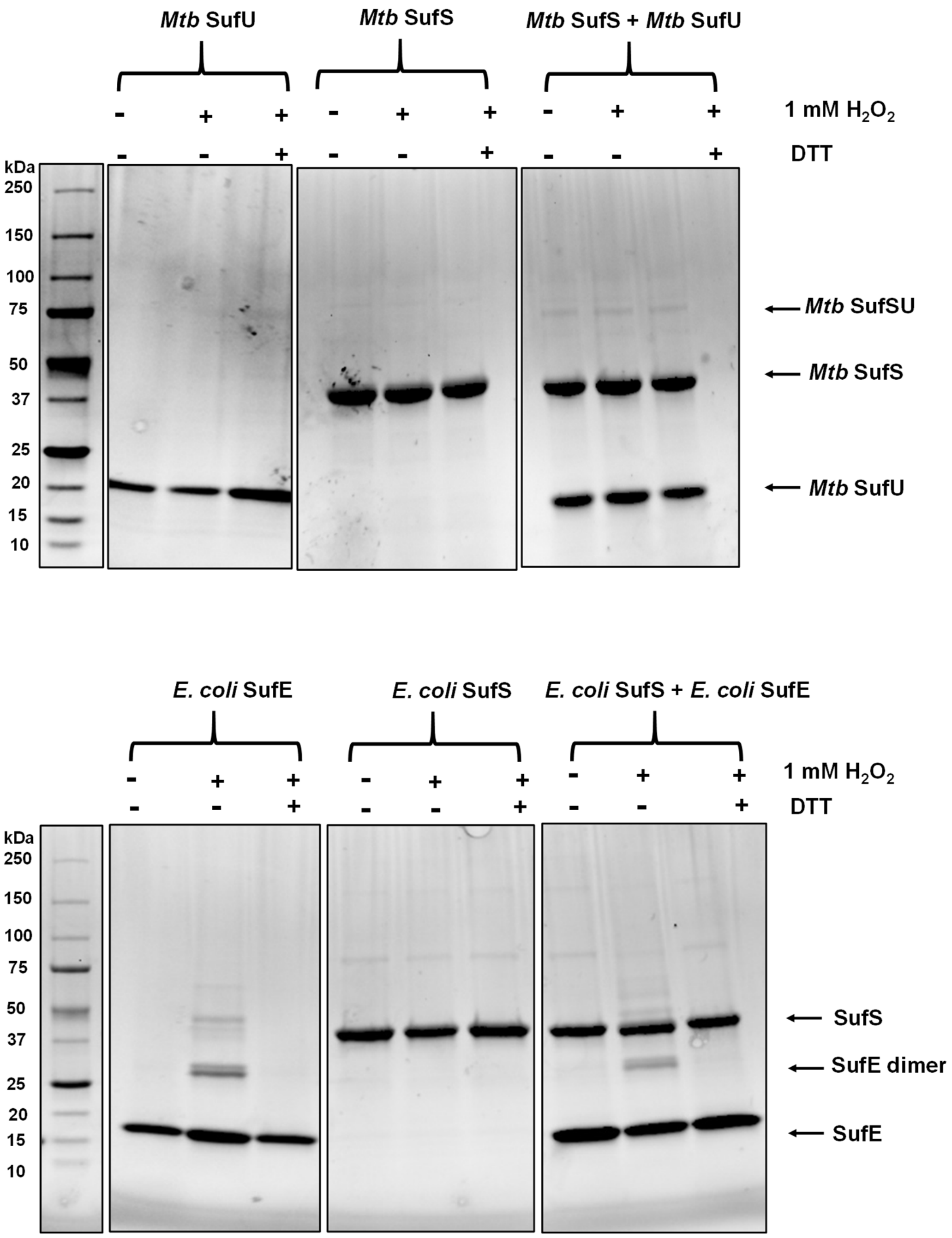

Figure S8. SDS-PAGE gel of H_2O_2 treated SufS and SufU proteins . SDS-PAGE gels of *Mtb* SufS, SufU, SufS-SufU **(A)** as well as *E. coli* SufS, SufE and SufS-SufE **(B)** in presence of H_2O_2 with or without DTT. The gel shows control proteins without any treatment (first lane of each category), proteins treated with 1 mM H_2O_2 for 30 mins (second lane of each category), and treated proteins with H_2O_2 (30 mins) and 2 mM DTT (third lane of each category). All samples were then dissolved in 1XSDS loading buffer not containing any reducing agents and were heated at 95°C for 15 min. and loaded on the gel. SufE was found to be sensitive to H_2O_2 exposure as it tends to form disulfide bridges since dimer (34%) and tetramer (6%) species were detected on gel that disappeared upon treatment with DTT.

Table S1. X-Ray data and refinement statistics.

**Data collection statistics** (from the XDS CORRECT file)	
Beamline	ESRF-ID30B
Wavelength (Å)	0.9763
Temperature (K)	100
Space group	*P1*
a, b, c (Å)	62.3, 75.6, 75.6
α, β, γ (°)	92.4, 109.6, 99.7
Mosaicity (°)	0.166
Resolution range (Å)	50-1.65 (1.75-1.65)§
Total No of reflections	422116 (64985)§
No of unique reflections	147275 (22934)§
Wilson B factor (Å^2)	29.1
Completeness (%)	95.7 (92.4)§
Multiplicity	2.9 (2.8)§
Mean I/σ(I)	7.47 (0.96)§
R_{meas} (%)	10.4 (117.6)§
CC1/2 (%)	99.6 (54.1)§
**Refinement statistics** (from the final refmac log file)	
PDB code of the final refined model	8ODQ
PDB code for intial Molecular	5XT5
No of molecules SufU-SufS in A.U	2
No. of atoms refined	9784
Resolution range (Å)	48.7-1.65Å
No of reflections use for refinement	140036
Refinement Rwork/Rfree (%)	17.9/21.4
Mean B value (Å^2)	27.0
RMSD bond length (Å)	0.01
RMSD bond angle (°)	2.1

§ : in parenthesis, values of high resolution shell

Table S2. Contact interactions (amino-acid involved).

	SufU	SufS	Distance (Å)
**A. Hydrogen bonds**	C:SER 68 [OG]	D:ASP 355 [OD1]	2.87
	C:ARG 128 [NH2]	D:ASP 355 [OD1]	3.12
	C:TYR 9 [OH]	D:ASP 355 [OD1]	2.59
	C:SER 68 [N]	D:ASP 355 [OD2]	2.94
	C:ARG 128 [NH1]	D:GLN 358 [OE1]	2.98
	C:CYS 40 [S2]	D:VAL 369 [O]	3.29
	C:CYS 40 [S2]	D:HIS 371 [N]	3.11
	C:CYS 40 [S2]	D:ARG 368 [NE]	2.84
	C:CYS 40 [O]	D:HIS 354 [NE2]	2.83
	C:GLY 41 [O]	D:HIS 352 [NE2]	2.59
**B. Salt Bridges**	C:ARG 128 [NH2]	D:ASP 355 [OD1]	3.12
	C:ARG 128 [NH1]	D:ASP 355 [OD1]	3.92
	C:GLU 6 [OE2]	D:ARG 409 [NH1]	3.53
	C:ASP 42 [OD1]	D:HIS 352 [NE2]	3.33
	C:ASP 42 [OD1]	D:HIS 354 [ND1]	3.33
	C:ASP 42 [OD2]	D:HIS 354 [ND1]	3.19

	SufU		SufS	
	Residues	BSA(Å^2)	Residues	BSA(Å^2)
**C. Interfacing residues**	C:ARG 4	57.72	D:ARG 25	76.45
	C:LEU 5	59.63	D:GLY 26	17.27
	C:GLU 6	62.27	D:GLY 350	10.91
	C:ILE 8	5.69	D:VAL 351	1.18
	C:LEU 14	35.98	D:VAL 359	12.38
	C:TYR 17	39.12	D:ASP 362	44.81
	C:LYS 18	44.87	D:ARG 368	15.94
	C:ILE 39	28.72	D:GLY 370	15.31
	C:ASP 42	7.20	D:ALA 384	44.38
	C:GLN 65	34.10	D:ALA 385	4.18
	C:GLY 66	9.66	D:ARG 409	37.55
	C:CYS 67	34.94	D:PHE 413	96.72
	C:LYS 124	27.41	D:PHE 414	71.20
	C:TYR 125	52.87	D:GLY 415	1.57
	C:PRO 126	4.36	D:ARG 416	21.40
	C:CYS 131	2.17		

Table S2 : PISA calculation [1] performed on the complex *Mtb* SufS-SufU (subunit C and subunit D, Figure S5). **A.** Hydrogen bonds listed with a cut-off of 3.3 Å. **B.** Salt bridges listed with a cut-off of 4 Å. **C.** Other interfacing residues between SufU and SufS that do not form hydrogen bonds or salt bridges. **BSA** is the residue Buried Surface Area upon SufS-SufU complex formation.

References:

1. Krissinel, E.; Henrick, K. Inference of macromolecular assemblies from crystalline state. *J. Mol. Biol.* **2007,** *372* (3), 774-97.
